# The Effect of Fibrates on Kidney Function and Chronic Kidney Disease Progression: A Systematic Review and Meta-Analysis of Randomised Studies

**DOI:** 10.3390/jcm11030768

**Published:** 2022-01-31

**Authors:** Alexandros Hadjivasilis, Panayiotis Kouis, Andreas Kousios, Andrie Panayiotou

**Affiliations:** 1 Cardiovascular Epidemiology and Genetics Research Lab, Cyprus International Institute for Environmental and Public Health, Cyprus University of Technology, Limassol 3036, Cyprus; ahadjivasilis@gmail.com (A.H.); kouis.panayiotis@ucy.ac.cy (P.K.); andrie.panayiotou@cut.ac.cy (A.P.); 2 Respiratory Physiology Laboratory, Medical School, University of Cyprus, Nicosia 1678, Cyprus; 3 West London Renal and Transplant Centre, Hammersmith Hospital, Imperial College Healthcare NHS Trust, Du Cane Road, London W12 0HS, UK; 4 Centre for Inflammatory Disease, Imperial College London, London W12 0HS, UK

**Keywords:** cardivacular disease, renal function, proteinuria, fibrates

## Abstract

Aim: Fibrates have proven efficacy in cardiovascular risk reduction and are commonly used, in addition to statins, to control hypertriglyceridaemia. Their use is often limited due to reduction in glomerular filtration rate at treatment initiation. However, recent studies suggest benign changes in kidney function and improvement of proteinuria, an established early marker of microvascular disease and kidney disease progression. We summarize the evidence from existing trials and provide a summary of effects of fibrates, alone or in combination, on kidney disease progression and proteinuria. Methods and Results: Systematic review and meta-analysis of randomized, controlled trials (PROSPERO CRD42020187764). Out of 12,243 potentially eligible studies, 29 were included in qualitative and quantitative analysis, with a total of 20,176 patients. Mean creatinine increased by 1.05 (95% CI (0.63 to 1.46)) units in patients receiving fibrates vs. comparator, and this was similar in all other subgroups. eGFR showed a bigger decrease in the fibrates arm (SMD −1.99; 95% CI (−3.49 to −0.48)) when all studies were pooled together. Notably, short-term serum creatinine and eGFR changes remained constant in the long-term. Pooled estimates show that fibrates improve albuminuria progression, RR 0.86; 95% CI (0.76 to 0.98); albuminuria regression, RR 1.19; 95% CI (1.08 to 1.310). Conclusions: Fibrates improve albuminuria in patients with and without diabetes when used to treat hyperlipidaemia. The modest creatinine increase should not be a limiting factor for fibrate initiation in people with preserved renal function or mild CKD. The long-term effects on kidney disease progression warrant further study.

## 1. Introduction

Chronic kidney disease (CKD) is a common disease with increasing prevalence. More than 20 million Americans are affected, with approximately 500,000 of them being diagnosed with end stage kidney disease (ESKD) [1]. Patients with CKD are at increased risk of developing cardiovascular disease (CVD) which is the leading cause of death in this population [2]. CVD mortality accounts for up to 50% of deaths in patients who progress to ESKD [3].

Fibrates, which are peroxisome proliferator-activated receptor (PPAR) a-activators, are agents used for the treatment of dyslipidaemia. Specifically, they lower triglyceride and low-density lipoprotein (LDL) levels, while they increase high density lipoprotein (HDL) levels [4]. Patients with CKD have a distinct lipid profile characterised by elevated triglyceride-rich lipoproteins and low HDL levels, which are associated with subclinical atherosclerosis, coronary artery disease and mortality [5].

Until recently, data from various studies had raised concerns that fibrates might have nephrotoxic effects because of the increase in serum creatinine levels and the decrease in glomerular filtration rate (GFR) when fibrates were administered. Although these initial changes in creatinine levels and estimated GFR are true, newer data from well-designed randomised controlled trials (RCTs) show that these changes do not affect the actual kidney function [6,7,8]. In fact, the FIELD [6], the ACCORD [7] and the DAIS [8] trials showed a beneficial effect of fibrates on albuminuria in diabetic patients; an established marker of early microvascular disease and a predictor of adverse CVD outcomes and kidney disease progression.

A meta-analysis previously examined the effect of fibrates on CVD outcomes in people with kidney disease demonstrating efficacy and safety in this patient group [9]. However, the authors did not focus on long-term renal outcomes and did not take into account fibrate–statin co-administration; a common practice in patients with dyslipidaemia. Although the effect of co-administration was examined by Guo et al. [10], their study did not examine the combined effect of the two drugs in kidney disease. Therefore, little is known about the combined effect of the two drugs in kidney disease and kidney disease progression in such patients. Moreover, a quantitative estimate of the change in kidney function expected with fibrate initiation in the short and long-term is unknown. More importantly, the effect of fibrates on kidney disease progression has not been systematically studied in patients at risk of CKD or those with established CKD.

The objective of this systematic review and meta-analysis was to summarize evidence from existing trials and provide a summary of effects of fibrates, alone or in combination treatment, on kidney disease and kidney disease progression. The primary endpoints examined in our study were: (a) the effect of fibrates on serum creatinine and kidney function, (b) their effect on proteinuria or albuminuria and (c) their effect on development of ESKD.

## 2. Methods

The study was conducted in accordance with the guidelines from the Preferred Reporting Items for Systematic reviews and Meta-analyses (PRISMA) statement [11].

### 2.1. Protocol and Registration

A predefined protocol was drafted and registered in the Prospective Register of Systematic Reviews (PROSPERO) with registration number CRD42020187764; it can also be found as a publication in a pre-prints platform [12].

### 2.2. Eligibility Criteria

We developed a PICOS model to predefine our inclusion criteria. PICOS stands for population, intervention, comparator, outcome and study type. Specifically, we included adult patients (18 years or older) with chronic kidney disease (eGFR < 60 mL/min) or patients with risk factors for CKD. The intervention was fibrates alone or fibrates plus statin administration with placebo as the comparator in the former and statin in the latter. The outcomes of interest were changes in serum creatinine, renal function (using any GFR estimation formula or measured GFR), CKD progression (GFR loss of more than 5 mL/min per year), development of ESKD, change in proteinuria, development of proteinuria and proteinuria reduction. Progression of albuminuria is defined as a change from normal urine albumin excretion to microalbuminuria or macroalbuminuria or from microalbuminuria to macroalbuminuria. Albuminuria regression is defined as a change from macroalbuminuria or microalbuminuria to normal urine albumin excretion or from macroalbuminuria to normal urine albumin excretion. All included studies were randomised controlled trials.

The exclusion criteria were paediatric patients (younger than 18 years old) or any form of renal replacement therapy (peritoneal dialysis or haemodialysis) transplantation or eGFR < 15 mL/min/1.73 m^2^.

### 2.3. Information Sources and Search

MEDLINE, SCOPUS, the Cochrane Library and the Clinical Trials registers (clinicaltrials.gov (accessed on 1 October 2020)); (clinicaltrialsregistry.eu (accessed on 1 October 2020)) electronic databases were searched from inception until July 2020. A combination of relevant Medical Subject Headings (MeSH) terms and relevant short terms were used as keywords with a modified algorithm in each database. An example of the search syntax can be found in the supplementary file.

### 2.4. Study Selection and Data Collection Process

Two independent reviewers (AH and AK) screened all potentially eligible studies against the inclusion and exclusion criteria and any disagreement was resolved by a third reviewer (AP).

After the screening, extraction of data from all included studies, was done by AH and AK independently in a predefined excel sheet. Data extraction was done for study main characteristics, design, methodology, sample characteristics, intervention, comparator group characteristics and outcomes as listed earlier in the PICOS model.

### 2.5. Risk of Bias

Two independent reviewers (AH and PK) assessed all included studies for risk of bias using the Cochrane tool for assessing the risk of bias in RCTs [13]. Again, any disagreement was resolved by a third reviewer (AP).

### 2.6. Summary Measures and Synthesis of Results

Characteristics of included studies were summarised on tables and presented in the form of narrative synthesis in the text. Suitability of studies for inclusion in the meta-analysis was based on clinical, methodological and statistical homogeneity. Where meta-analysis was possible, we analysed data accordingly:

- For continuous outcomes (i.e., eGFR, creatinine), we performed a generalised inverse variance analysis of standardised mean difference between patients in intervention and control group, pre and post administration of intervention/treatment/placebo using a random effects model.
- For categorical outcomes, relative risk was calculated using number of affected patients per outcome of interest from the included studies and a pooled estimate is presented using forest plots. Pooled estimates were calculated with a random-effects model (Der Simonian–Laird method) to account for both within and between study variability. Heterogeneity between synthesised studies were calculated using the I^2^ statistic and the presence of publication bias was investigated graphically by precision funnel plots. All statistical analyses were performed using STATA (Version 14, StataCorp, College Station, TX, USA).

## 3. Results

### 3.1. Study Selection

**Figure 1:**
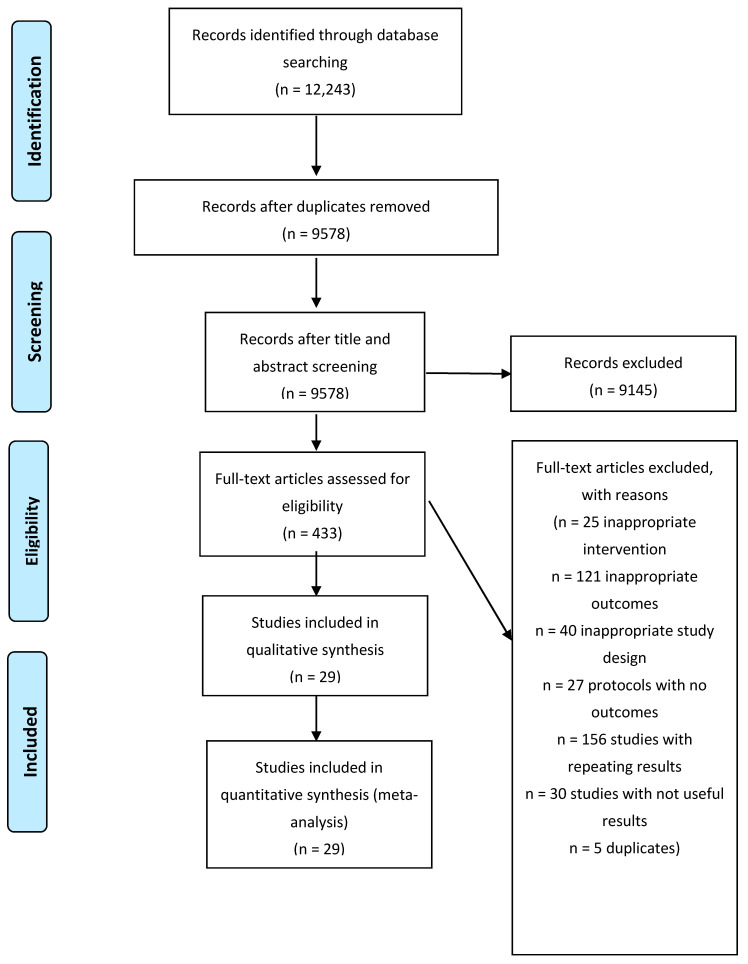
Flow diagram.

A total of 12,243 potentially eligible studies were identified using the predefined search algorithm(s). After duplicates removal, the title and abstract of the remaining 9578 studies were screened and 9145 studies were further excluded. The remaining 433 studies were assessed for eligibility and 404 were excluded with reasons as presented in Figure 1. A total of 29 studies [7,8,14,15,16,17,18,19,20,21,22,23,24,25,26,27,28,29,30,31,32,33,34,35,36,37,38,39] were included in the qualitative and the quantitative analysis.

### 3.2. Study Characteristics

A total of 20,176 patients were included in the eligible studies, 10,249 in the intervention arm and 9841 in the comparator arm while 86 patients received both interventions in the setting of crossover studies. The majority of the studies (*n* = 21) had examined fenofibrate alone or in combination [7,8,14,15,17,18,20,21,22,24,25,26,27,28,29,30,31,33,34,35,40,41] and 11 of them fenofibrate vs. placebo [8,14,15,17,18,26,27,31,34,35]. Among the included studies more than half of the participants came from the FIELD and the ACCORD studies. Study characteristics are summarised in Table 1.

### 3.3. Risk of Bias within Studies

Risk of bias was assessed with the Cochrane tool [13] for assessing risk of bias in randomised control trials and the results can be seen in Table 2 and Table 3. As expected, because of the strict methodology RCTs have to follow, the majority of studies had low risk of bias in most of the domains. Some studies examined the results without blinding the assessors and this was considered a high risk of bias. Also, when not enough information was provided in the manuscript, some domains were characterised as not clear.

### 3.4. Synthesis of Results

Standardised mean differences were used to assess the effect of continuous data and relative risk to assess outcomes that were reporting number of patients. All forest plots were done using the random effects models. A summary of all pooled effect estimates can be found in Table 4.

### 3.5. Creatinine Change

**Figure 2:**
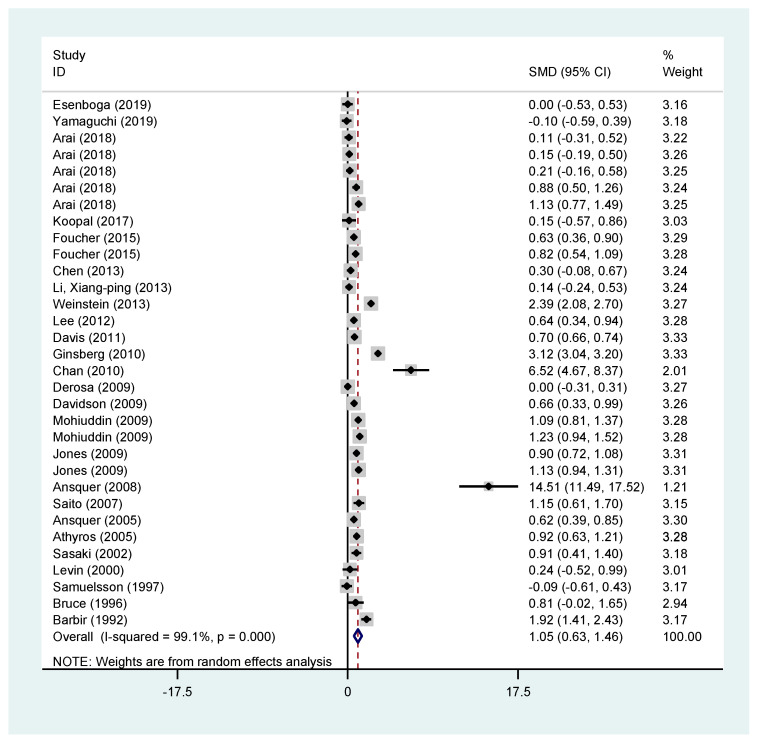
Pooled effects of creatinine from all included studies.

Creatinine appears to have an increase of 1.05 (95% CI (0.63–1.46)) mg/dL when comparing the mean change of patients receiving fibrates vs. the mean change of patients receiving the comparator. In this analysis the comparison was made for studies using either fibrates alone vs. placebo or fibrates plus statins vs. that statin. The results are presented in Figure 2.

Creatinine seems to increase in all arms where fibrates were used, whether this was fenofibrate (all studies: standardised mean difference (SMD) 1.34; 95% CI (0.82–1.86), fenofibrate vs. placebo: SMD 1.22; 95% CI (0.74–1.89), fenofibrate plus statin vs. statin: SMD 1.07; 95% CI (0.34–1.79)) or bezafibrate vs. a comparator, SMD 0.68; 95% CI (0.01–1.34). Results can be found in the supplementary file.

Creatinine change was also assessed in patients with diabetes with similar results (all studies SMD 1.49 95% CI (0.29–2.71) and in studies examining fenofibrates vs. placebo: SMD 0.86; 95% CI (0.35–1.37)). Results can be found in the supplementary file.

Similarly, the short-term effect (3 months or less) of fibrates in creatinine change was examined. For all studies, the SMD of creatinine in the fibrate groups was 0.97 95% CI (0.67–1.26). For studies using fibrates alone the SMD was 1.23 95% CI (0.88–1.58), whilst for studies examining fenofibrate vs. placebo the SMD was 2.73 95% CI (1.53–3.94) and for studies examining the effect of the combination of fenofibrate and a statin 1.02 (0.70–1.34). Short-term effect of creatinine change in studies examining bezafibrates was not statistically significant (all studies: SMD 0.65 95% CI (−0.11 to 1.42) and bezafibrate vs. placebo: SMD 0.79 95% CI (−0.17 to 1.75)). However, all the above results had significant heterogeneity. Results can be found in the supplementary file.

### 3.6. eGFR

**Figure 3:**
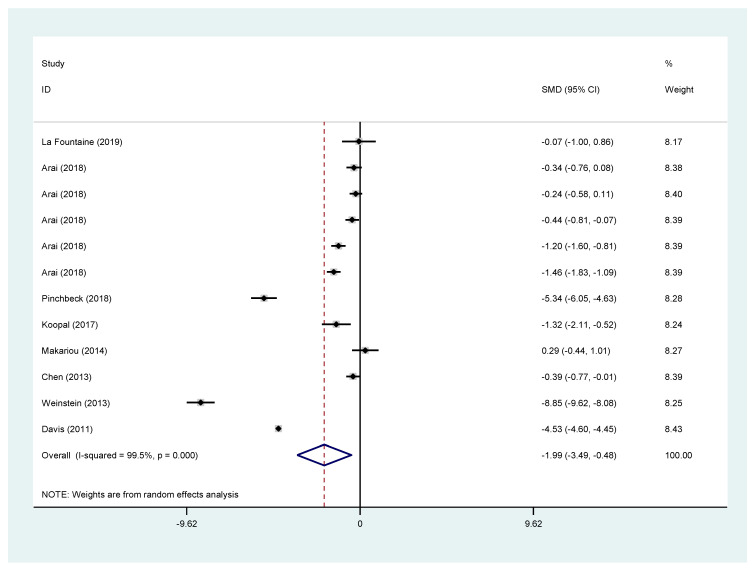
Pooled effects for eGFR change from all included studies.

eGFR showed a bigger decrease in the fibrates arm (SMD −1.99; 95% CI (−3.49 to −0.48)) when all studies were pooled together. The same appears for all the subgroup analyses [fenofibrate vs. placebo or fenofibrate plus statin vs. statin: SMD −2.69; 95% CI (−4.47 to −0.91), fenofibrate alone vs. placebo: SMD −2.53; 95% CI (−4.46 to −0.60), fenofibrate plus statin vs. statin: SMD −2.98; 95% CI (−8.00 to 2.05)] with only the latter being statistically non-significant. The results are presented in Figure 3.

Similarly, the short-term effect (3 months or less) of fibrates in eGFR change was examined. For all studies, the SMD of eGFR in the fibrate groups was −1.88 95% CI (−3.02 to −0.73). For studies using fenofibrate the was SMD −2.64 95% CI (−4.55 to −0.72), for studies examining fenofibrate vs. placebo −2.38 95% CI (−4.20 to −0.57) and for studies examining the effect of the combination of fenofibrates and statins −2.98 95% CI (−8.00 to 2.05) with the latter being statistically not significant. Likewise, all these results were affected by significant heterogeneity. Results can be found in the supplementary file.

### 3.7. Albuminuria

Data reporting on albuminuria were analysed as progression, regression and urinary albumin excretion mean changes.

**Figure 4:**
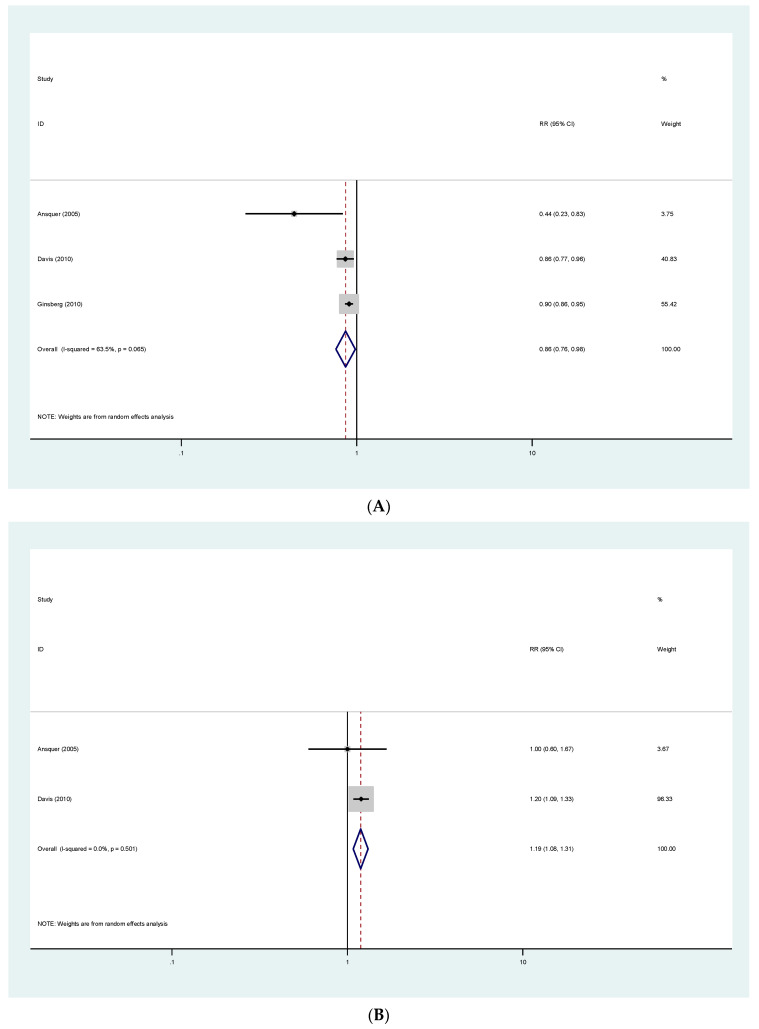
(**A**) Effects in proteinuria progression. (**B**) Effects in proteinuria regression.

Pooled estimates show that patients receiving fibrates were less likely to have albuminuria progression (RR 0.86; 95% CI (0.76–0.98)) and more likely to have albuminuria regression (RR 1.19; 95% CI (1.08–1.31)). Results are presented in Figure 4A,B.

### 3.8. Chronic Kidney Disease and End Stage Kidney Disease

Most of the studies included patients with normal kidney function. Only one study, focused exclusively on patients with CKD stage III [24] showing that a combination of fenofibric acid and a statin was safe in these patients for at least 16 weeks. In addition, two studies included patients with range of eGFR 10–70 mL/min/min, showing that GFR remained similar between patients receiving fenofibrate and patients receiving the control and that urinary protein excretion did not change after 12 months of follow up [36]. A result confirmed also by Levin et al. [35] where despite the initial increase in the serum creatinine, the change from baseline of creatinine for the two groups was similar and protein excretion was also similar six months after, at the end of the study.

Data from the available two studies [26,36] show some evidence that fibrates might reduce ESKD progression, but this was not statistically significant (RR 0.85; 95% CI (0.49–1.49)), perhaps due to the small number of studies/participants. Results can be found in the supplementary file.

### 3.9. Publication Bias across Studies

The presence of publication bias was investigated graphically by precision funnel plots and it can be found in the supplementary file. In general, many of the studies failed to identify precisely the pooled effect estimated that we calculated in this meta-analysis. There was no evidence of large publication bias for any of the outcomes studied.

## 4. Discussion

Fibrates are commonly used drugs in patients with CVD with established results [42]. However, the effects of these drugs in kidney function, kidney disease progression and in patients with established CKD are not well examined. Their use is often limited due to concerns of nephrotoxicity.

In this systematic review and meta-analysis, we provide information about the safety of fibrates when used alone or in combination with statins. This adds to the previous meta-analysis by Min Jun et al. [9] which examined mainly the effect of fibrates on CVD and reported the effects of fibrates when used as a single treatment.

Additionally, our results show that both short-term (3 months or less) and long-term creatinine change have similar values, therefore creatinine might have a rise initially, but remains relatively constant afterwards. Similarly, short term changes in eGFR remain relatively similar with long-term use of fibrates.

Even though fibrates administration results in an increase in serum creatinine, the fact that progression of albuminuria is reducing [6,7,8] and regression is increasing [6,8] is reassuring. We cannot comment on the effect of fibrates on ESKD development [26,36] as our data do not support statistically significant effects. It is important to note the relatively short follow-up of studies. It is also worth mentioning that, creatinine elevation was fully reversible in the FIELD study [6] eight weeks after the completion of the trial but with sustained CVD benefits, and eGFR returned to baseline values in the ACCORD study [7] after fenofibrate discontinuation. Both studies did not demonstrate any added major adverse event compared to the control. In addition, in a subgroup of participants in the ACCORD trial, Chauhan et al. demonstrated that the rise in serum creatinine is not accompanied by an increase of urinary biomarkers representing tubular injury, inflammation or fibrosis, providing further support for benign change in kidney function [43].

The mechanism by which serum creatinine is increased once fibrates are administered is not yet fully understood. In part, it can be explained by the involvement of the peroxisome proliferator receptors, that generate vasodilatory prostaglandins resulting in an increase in kidney blood filtration which increases serum creatinine and decreases GFR [44]. Moreover, emerging evidence, suggest that alterations in the PPAR pathway at the molecular level are both early and late events in CKD and CKD progression mouse models [45,46] and fenofibrate treatment exerts nephroprotective effects thought the attenuation of inflammatory and fibrotic pathways [41,47,48]. Enhancing binding at the PPARa receptor is a promising development, influencing downstream gene and physiological effects of PPRAa activation. The peroxisome proliferator-activated receptor alpha modulator pemafibrate has a structure which increases its selectivity for PPRAa and consequently its potency by >2500-fold, compared to fenofibrate, and has a better benefit–risk profile. The results of the PROMINENT study, a phase III placebo-controlled RCT, on the effect of pemafibrate on CVD events in high-risk type 2 diabetics, including those with mild to moderate renal impairment are awaited (ClinicalTrials.gov: NCT03071692).

In our meta-analysis, the most commonly used fibrate was fenofibrate. Fenofibrates were found to be effective and safe in the FIELD study, even for patients with reduced eGFR [49]. In fact, patients with eGFR of 30–59 mL/min/1.73 m^2^ were the ones with the greatest CVD reduction and at the same time, no further ESKD progressions were observed. This effect was also observed in the VA-HIT trial [50] where fibrates were effective in reducing CVD in patients with mild to moderate renal insufficiency.

Furthermore, because of the CVD risk improvement fibrates induce, it is possible that the reason behind the preservation of kidney function eventually, and the reduction in the progression of microalbuminuria, is attributed to the benefit they have at the vascular level, at least in patients with type 2 diabetes [8].

Although, we found high heterogeneity and publication bias in pooled estimates relating to kidney function, the effect of fibrates in progression and regression of proteinuria, two of the main outcomes of our study, had 63.5% and 0% heterogeneity, respectively. These were analyses with 14,385 and 2152 patients accordingly, therefore we decided to proceed to a meta-analysis, providing robust quantitative estimates on the effect of fibrates on albuminuria, a marker commonly used as a surrogate outcome for kidney disease progression and early CVD.

### Limitations

We observed large heterogeneity within and between the studies on eGFR and creatinine. Nonetheless, the direction of the effect estimate is in keeping with a priori expectation. An explanation for this heterogeneity is that almost all the included studies were not examining kidney related variables as a primary outcome, therefore the information obtained was related to patients with different kidney status and characteristics. Furthermore, different fibrates and different statins were used in the included studies even with sometimes different dosages, which could also be a reason for this heterogeneity. In addition, since kidney related outcomes were not the primary endpoint in most of the studies, the small number of participants in many of the studies introduced power issues within each study estimate. Additionally, the observation period for most of the studies was less than three months which might also have added to this heterogeneity. Thus, large multicentred randomised controlled trials are still needed to examine this topic, recruiting patients with similar kidney statuses. Last but not least, the effect of fibrates in patients with eGFR less than 30 was not examined so results cannot be extrapolated to this population.

## 5. Conclusions

Our study is in agreement with a previous meta-analysis and expands further on the effect of fibrates alone, or in combination with a statin, on kidney function and proteinuria. The modest increase in creatinine at treatment initiation remains unchanged throughout the treatment course and is reversible upon cessation of fibrate treatment. Patients with CKD are commonly older people presenting with co-existing diabetes and a distinct lipid profile phenotype which renders fibrates a promising agent to study for the treatment of CVD and kidney disease progression in kidney disease patients. Data on the safety of fibrates in CKD are lacking. Importantly, our analysis provides evidence that fibrates not only reduce albuminuria progression but also increase albuminuria regression in patients with and without diabetes when used to treat hyperlipidaemia. Longer-term studies are needed on the effect of fibrates in delaying ESKD.

## Figures and Tables

**Table 1:** Study characteristics of included studies.

First Author	Year	Study Name	Intervention	Control	No Intervention	No Control
Esenboga	2019		fenofibrate 250 mg/d	placebo	30	26
La Fountaine	2019		fenofibrate 145 mg	control	10	8
Yamaguchi	2019		bezafibrate 400 mg	eicosapentaenoic acid 1.8 g/day	33	31
Arai	2018		pemafibrate (0.1 or 0.2 or 0.4 mg) or fenofibrate (100 mg or 200)	placebo	pemafibrate 0.1 mg: 45, pemafibrate 0.2 mg: 128, pemafibrate 0.4 mg: 84, fenofibrate 100 mg: 85, fenofibrate 200 mg: 140	43
Pinchbeck	2018	FAME	145 mg fenofibrate	placebo	70	70
Koopal	2017		bezafibrate	placebo	15 in total	crossover
Foucher	2015		fenofibrate/simvastatin 145/20 mg or 145/40 mg	simvastatin 20 mg or 40 mg	fenofibrate/simvastatin 145/20 mg: 109, fenofibrate/simvastatin 145/40 mg: 110	simvastatin 20 mg: 114, simvastatin 40 mg: 112
Makariou	2014		add-on-statin micronised fenofibrate (200 mg)	rosuvastatin 40 mg	13	17
Chen	2013		fenofibrate 80 mg + rosuvastatin 5 mg	fosuvastatin 10 mg	50	62
Li Xiang-ping	2013		atorvastatin 20 mg + bezafibrate 200 mg	atorvastatin 20 mg	52	52
Weinstein	2013		fenofibric acid + rosuvastatin 5 then 10 mg	rosuvastatin 5 then 10	140	140
Lee	2012		rosuvastatin10 mg + fenofibrate160 mg	rosuvastatin10 mg	90	90
Davis	2011	FIELD	fenofibrate	placebo	4895	4900
Ginsberg	2010	ACCORD	fenofibrate + simvastaatin	placebo + simvastatin	2765	2753
Chan	2010		fenofibrate (145 mg/day)	placebo	15 in total	crossover
Derosa	2009		fenofibrate 145 mg + simvastatin 40 mg/d	simvastatin 40 mg/d	79	82
Davidson	2009		atorvastatin 40 mg and fenofibrate 100 mg	atorvastatin 40 mg, or fenofibrate 145 mg	73	74 for statin
Mohiuddin	2009		fenofibric acid 135 mg+ rosuvastatin 20 mg OR fenofibric acids 135 mg + rosuvastatin 40 mg	rosuvastatin 20 mg OR rosuvastatin 40 mg	fenofibric acid 135 mg+ rosuvastatin 20 mg: 113, fenofibric acids 135 mg + rosuvastatin 40 mg: 111	rosuvastatin 20 mg: 116, rosuvastatin 40 mg: 112
Jones	2009		fenofibric acid 135 mg + rosuvastatin 10 mg OR fenofibric acids 135 mg + rosuvastatin 20 mg	rosuvastatin 10 mg OR rosuvastatin 20 mg	fenofibric acid 135 mg + rosuvastatin 10 mg: 261, fenofibric acids 135 mg + rosuvastatin 20 mg: 262	rosuvastatin 10 mg: 265, rosuvastatin 20 mg: 266
Ansquer	2008		fenofibrate (160-mg/	placebo	21 in total	crossover
Saito	2007		bezafibrate 200 mg	placebo	27	35
Ansquer	2005	DAIS	200 mg of micronised fenofibrate	placebo	155	159
Athyros	2005		fenofibrate 200 mg OR fenofibrate 200 mg + atorvastatin 20 mg	control (diet) OR atorvastatin, 20 mg/d	fenofibrate 200 mg: 100, fenofibrate 200 mg + atorvastatin 20 mg: 100	control (diet): 100, atorvastatin 20 mg: 100
Sasaki	2002		fenofibrate 300 mg	placebo	50 crossover	Data for creatinine from 35 patients
Levin	2000		fenofibrate	placebo	16	12
Samuelsson	1997		gemfibrozil	dietary	28	29
Bruce	1996		bezafibrate 400 mg	placebo	12	12
Barbir	1992		bezafibrate	placebo (maxepa (fish oil))	43	44
Jones	1990		bezafibrate (200 mg 3 times/day	placebo	20	17

**Table 2:** Risk of bias using for each included study.

Author	Year	Study Name	Random Sequence Generation (Selection Bias)	Allocation Concealment (Selection Bias)	Blinding of Participants and Personnel (Performance Bias)	Blinding of Outcome Assessment (Detection Bias)	Incomplete Outcome Data (Attrition Bias)	Selective Reporting (Reporting Bias)	Other Bias
Esenboga	2019		LOW	LOW	LOW	LOW	LOW	LOW	LOW
La Fountaine	2019		LOW	LOW	HIGH	HIGH	NOT CLEAR	LOW	NOT CLEAR
Yamaguchi	2019		LOW	LOW	HIGH	HIGH	LOW	LOW	LOW
Arai	2018		LOW	LOW	LOW	LOW	LOW	LOW	LOW
Pinchbeck	2018	FAME	LOW	LOW	LOW	LOW	LOW	LOW	NOT CLEAR
Koopal	2017		LOW	LOW	LOW	LOW	LOW	LOW	LOW
Foucher	2015		LOW	LOW	LOW	LOW	LOW	LOW	LOW
Makariou	2014		LOW	LOW	HIGH	HIGH	NOT CLEAR	LOW	NOT CLEAR
Chen	2013		LOW	LOW	HIGH	LOW	LOW	LOW	LOW
Li, Xiang ping	2013		LOW	LOW	HIGH	HIGH	LOW	LOW	LOW
Weinstein	2013		LOW	LOW	LOW	LOW	LOW	LOW	LOW
Lee	2012		LOW	LOW	HIGH	HIGH	LOW	LOW	NOT CLEAR
Davis	2011	FIELD	LOW	LOW	LOW	LOW	LOW	LOW	LOW
Ginsberg	2010	ACCORD	LOW	LOW	LOW	LOW	LOW	LOW	LOW
Chan	2010		LOW	LOW	LOW	LOW	LOW	LOW	LOW
Derosa	2009		LOW	NOT CLEAR	LOW	LOW	LOW	LOW	HIGH
Davidson	2009		LOW	LOW	LOW	LOW	LOW	LOW	LOW
Mohiuddin	2009		LOW	LOW	LOW	LOW	LOW	LOW	LOW
Jones	2009		LOW	LOW	LOW	LOW	LOW	LOW	LOW
Ansquer	2008		LOW	LOW	LOW	LOW	LOW	LOW	LOW
Saito	2007		LOW	LOW	LOW	LOW	LOW	LOW	LOW
Ansquer	2005	DAIS	LOW	LOW	LOW	LOW	LOW	LOW	LOW
Athyros	2005		LOW	LOW	HIGH	HIGH	LOW	LOW	LOW
Sasaki	2002		LOW	LOW	LOW	LOW	LOW	LOW	LOW
Levin	2000		LOW	LOW	LOW	LOW	LOW	LOW	LOW
Samuelsson	1997		LOW	LOW	HIGH	HIGH	LOW	LOW	LOW
Bruce	1996		LOW	LOW	LOW	LOW	LOW	LOW	NOT CLEAR
Barbir	1992		LOW	LOW	HIGH	HIGH	LOW	LOW	LOW
Jones	1990		LOW	LOW	LOW	LOW	LOW	LOW	LOW

**Table 3:** Risk of bias presented as percentage across all included studies.

	Low Risk	Not Clear	High Risk
Random sequence generation (selection bias)	100%	0%	0%
Allocation concealment (selection bias)	96.55%	3.45%	0%
Blinding of participants and personnel (performance bias)	68.97%	0%	31.03%
Blinding of outcome assessment (detection bias)	72.41%	0%	27.59%
Incomplete outcome data (attrition bias)	79.31%	20.69%	0%
Selective reporting (reporting bias)	100%	0%	0%
Other bias	79.31%	17.24%	3.45%

**Table 4:** Summary of effect estimates for each outcome.

Outcome	Method	Effect Estimate	95% CI Lower Limit	95% CI Upper Limit	Heterogeneity I^2^ %
Creatinine all studies	SMD	1.05	0.63	1.46	99.1
Creatinine studies using fenofibrate	SMD	1.34	0.82	1.86	99.4
Creatinine fenofibrate vs. placebo	SMD	1.22	0.74	1.89	94
Creatinine fenofibrate + statin vs. statin	SMD	1.07	0.34	1.79	99.3
Creatinine studies using bezafibrates	SMD	0.68	0.01	1.34	88.8
Creatinine bezafibrate vs. placebo	SMD	0.79	−0.01	1.59	88.9
Short term creatinine all studies	SMD	0.97	0.67	1.26	93.6
Short term creatinine studies using fenofibrate	SMD	1.23	0.88	1.58	94.1
Short term creatinine fenofibrate vs. placebo	SMD	2.73	1.53	3.94	96
Short term creatinine fenofibrate plus statin vs. statin	SMD	1.02	0.70	1.34	92.8
Short term creatinine studies using bezafibrate	SMD	0.65	−0.11	1.42	91
Short term creatinine bezafibrate vs. placebo	SMD	0.79	−0.17	1.75	91.7
Creatinine in patients with diabetes all studies	SMD	1.49	0.29	2.71	99.8
Creatinine in patients with diabetes, fenofibrate vs. placebo	SMD	0.86	0.35	1.37	91.8
eGFR all studies	SMD	−1.99	−3.42	−0.48	99.5
eGFR all studies with fenofibrates	SMD	−2.69	−4.47	−0.91	99.4
eGFR fenofibrate vs. placebo	SMD	−2.53	−4.46	−0.60	99.3
eGFR fenofibrate plus statin vs. statin	SMD	−2.98	−8.00	2.05	99.5
Short term eGFR all studies	SMD	−1.88	−3.02	−0.73	98.4
Short term eGFR studies using fenofibrate	SMD	−2.64	−4.55	−0.72	98.9
Short term eGFR fenofibrate vs. placebo	SMD	−2.38	−4.20	−0.57	97.8
Short term eGFR fenofibrate plus statin vs. statin	SMD	−2.98	−8.00	2.05	99.5
Progression of albuminuria	RR	0.86	0.76	0.98	63.5
Regression of albuminuria	RR	1.19	1.08	1.31	0
Urinary protein excretion change	SMD	−0.14	−0.56	0.29	0
End stage kidney disease development	RR	0.85	0.49	1.49	0

Abbreviations: CI—confidence interval, SMD—standardised mean difference, RR—relative risk.

## Data Availability

Not applicable.

## Supplementary Materials

The following supporting information can be downloaded at: https://www.mdpi.com/article/10.3390/jcm11030768/s1. Supplementary file: Pooled effects.
