# Supplementary material for: The Effect of Fibrates on Kidney Function and Chronic Kidney Disease Progression: A Systematic Review and Meta-Analysis of Randomised Studies
Source: J Clin Med. 2022 Jan 31;11(3):768. doi: 10.3390/jcm11030768 (PMC8836930; doi:10.3390/jcm11030768)
Supplement: Supplementary file 1 [file jcm-11-00768-s001.zip › jcm-1539652-supplementary.pdf]

Supplementary file

Page 3 - Figure S1: Pooled effects of creatinine from all studies using fenofibrates.

Page 4 - Figure S2: Pooled effects of creatinine in studies examining fenofibrates vs placebo.

Page 5 -Figure S3: Pooled effects of creatinine from studies examining fenofibrate + statin vs statin.

Page 6 -Figure S4: Pooled effects for creatinine from all studies in patients with diabetes

Page 7 -Figure S5: Pooled effects for creatinine in studies examining fenofibrate vs placebo in patients with diabetes

Page 8 -Figure S6: Pooled effects for creatinine in all studies examining Bezafibrate

Page 9 -Figure S7: Pooled effects for short term creatinine change (3 months or less) for all studies

Page 10 -Figure S8: Pooled effects for short term creatinine change for studies examining fenofibrates

Page 11 - Figure S9: Pooled effects for Short term creatinine changes for studies examining fenofibrate vs placebo

Page 12 - Figure S10: Pooled effects for short term creatinine changes for studies examining fenofibrate plus statin vs statin

Page 13 - Figure S11: Pooled effects for short term creatinine changes for studies examining Bezafibrates

Page 14 - Figure S12: Pooled effects for short term change for studies examining bezafibrate vs placebo

Page 15 - Figure S13: Pooled effects for eGFR for all studies examining fenofibrates

Page 16 - Figure S14: Pooled effects for eGFR for studies examining Fenofibrate vs placebo

Page 17 - Figure S15: Pooled effects for eGFR for studies examining Fenofibrate + statin vs statin

Page 18 - Figure S16: Pooled effects for short term eGFR changes

Page 19 - Figure S17: Pooled effects for short term eGFR change for studies examining fenofibrates

Page 20 - Figure S18: Pooled effects for short term eGFR change for studies examining fenofibrate vs placebo

Page 21 - Figure S19: Pooled effects for short term eGFR change for studies examining fenofibrate plus statin vs statin

Page 22 - Figure S20: Pooled effects for studies examining urinary protein excretion change

Page 23 - Figure S21: Pooled estimates for End stage kidney disease progression

Page 24 - Figure S22: Egger's precision publication bias plot for all studies examining creatinine changes.

Page 25 - Figure S23: Egger's precision publication bias plot for creatinine changes in studies using fenofibrates.

Page 26 - Figure S24: Egger's precision publication bias plot for creatinine changes studies examining fenofibrate + statin vs statin.

Page 27 - Figure S25: Egger's precision publication bias plot for short term creatinine change

Page 28 - Figure S26: Egger's precision publication bias plot for all studies examining change in eGFR

Page 29 – Table S1: Patient characteristics

Page 33 – Appendix A1 Search Algorithm

Figure S1: Pooled effects of creatinine from all studies using fenofibrates.

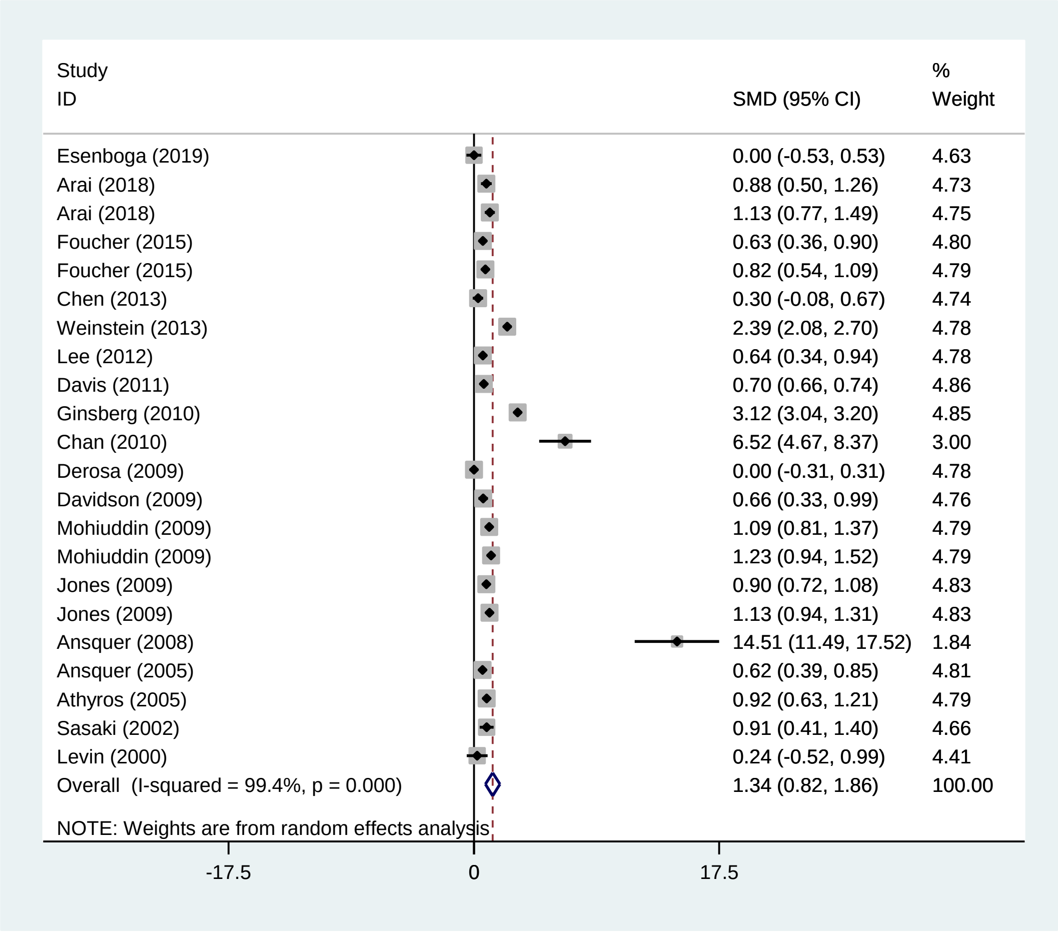

Figure S2: Pooled effects of creatinine in studies examining fenofibrates vs placebo.

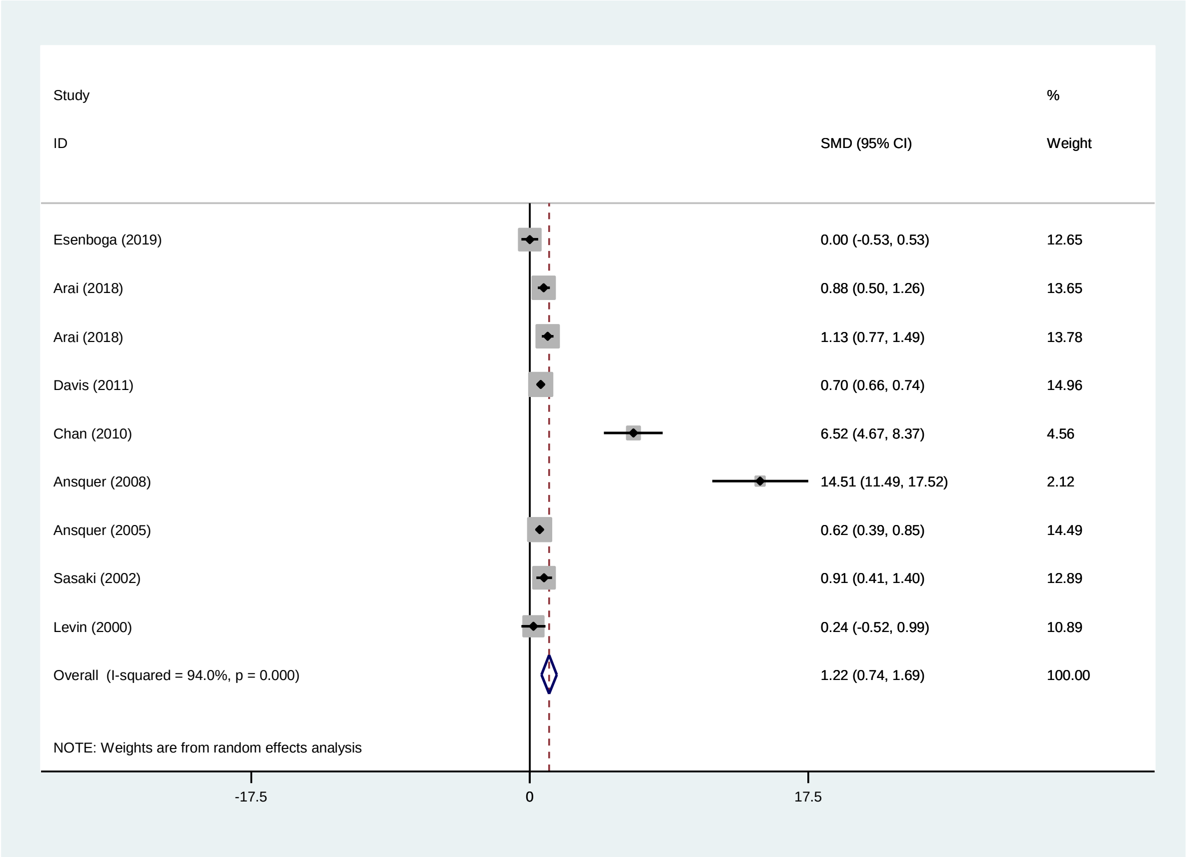

Figure S3: Pooled effects of creatinine from studies examining fenofibrate + statin vs statin.

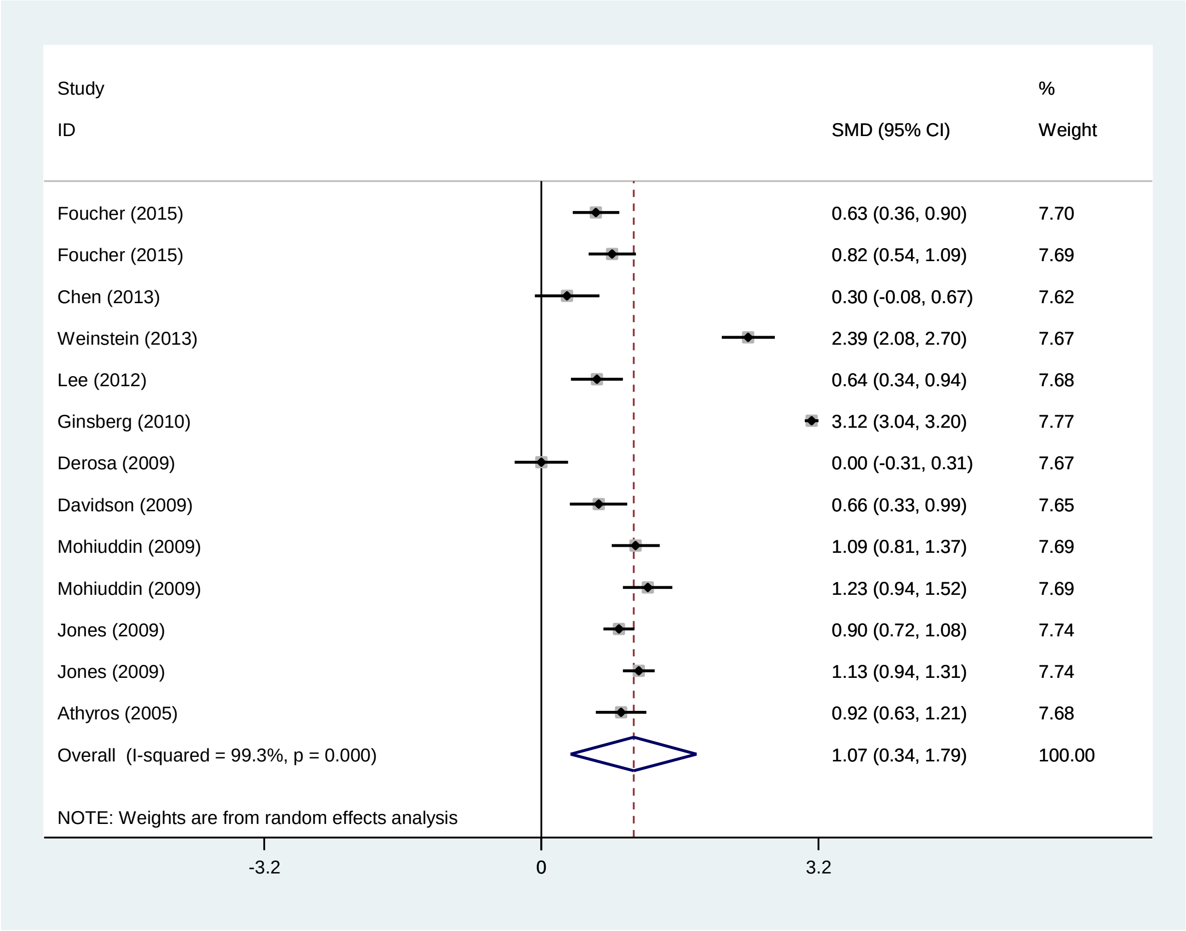

Figure S4: Pooled effects for creatinine from all studies in patients with diabetes

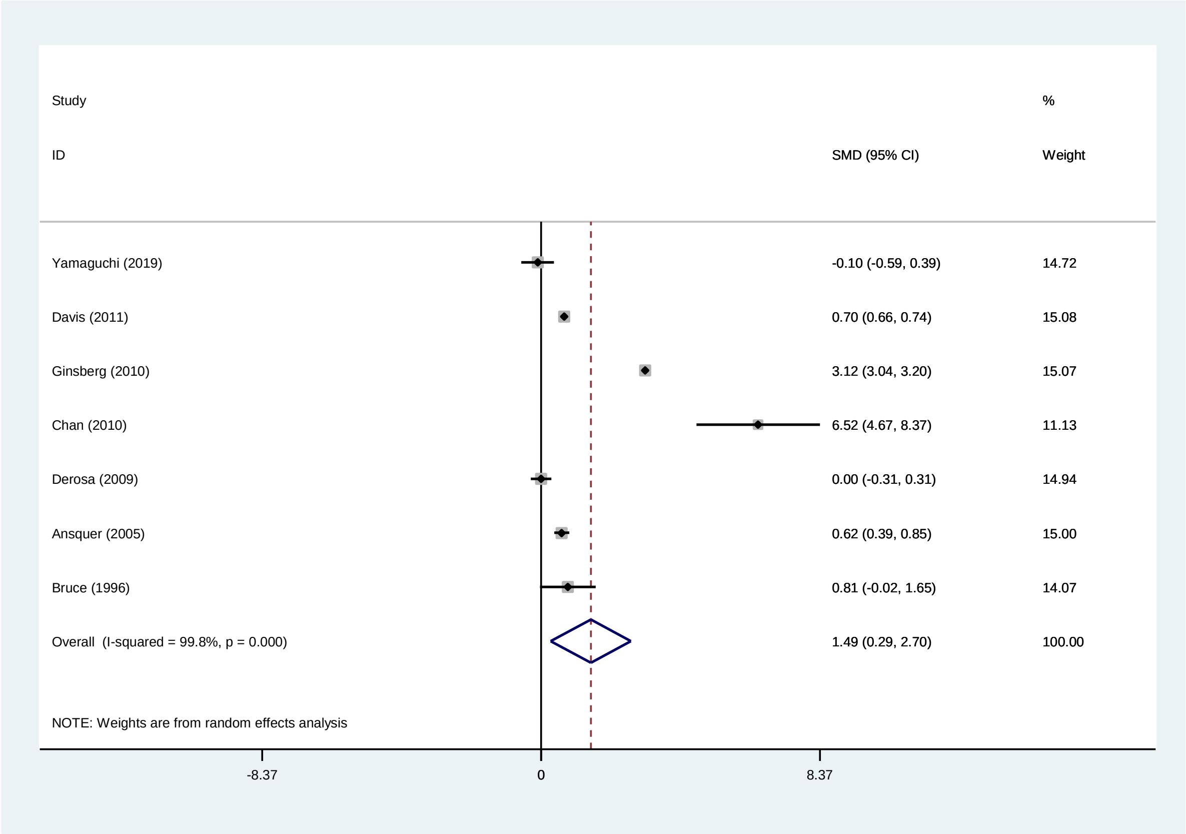

Figure S5: Pooled effects for creatinine in studies examining fenofibrate vs placebo in patients with diabetes

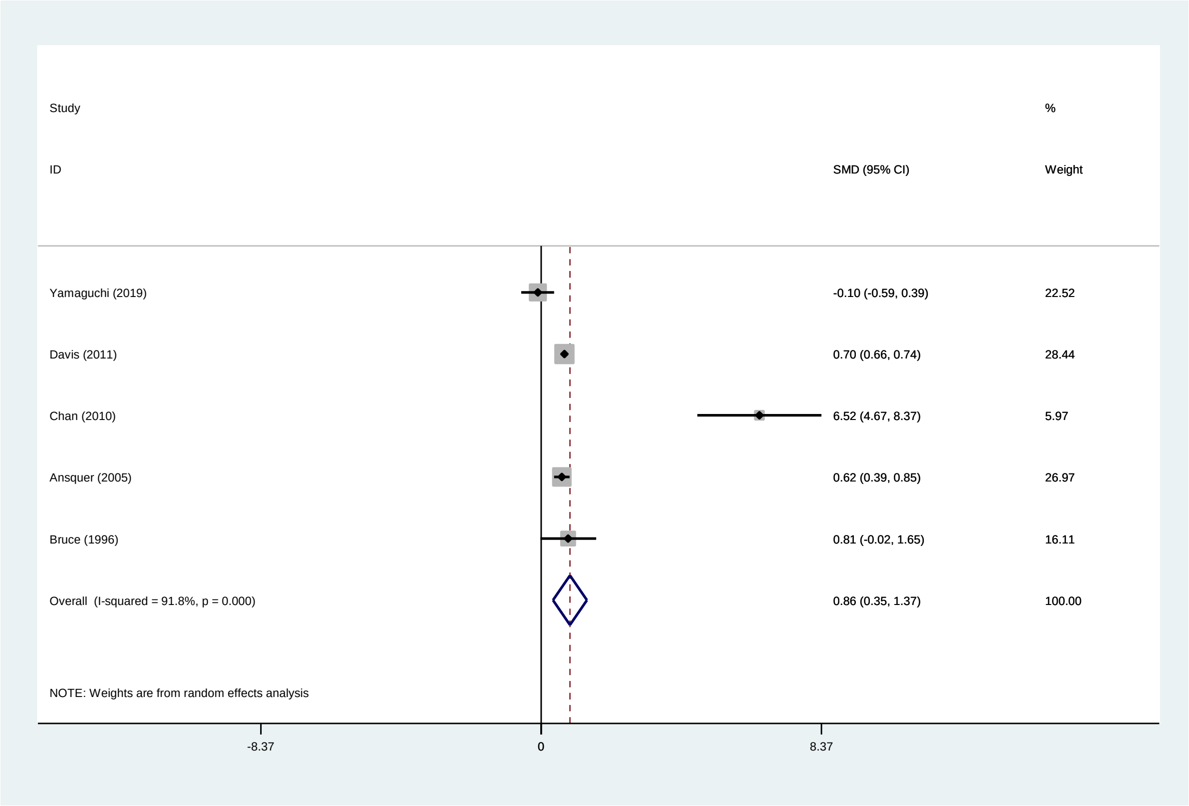

Figure S6: Pooled effects for creatinine in all studies examining Bezafibrate

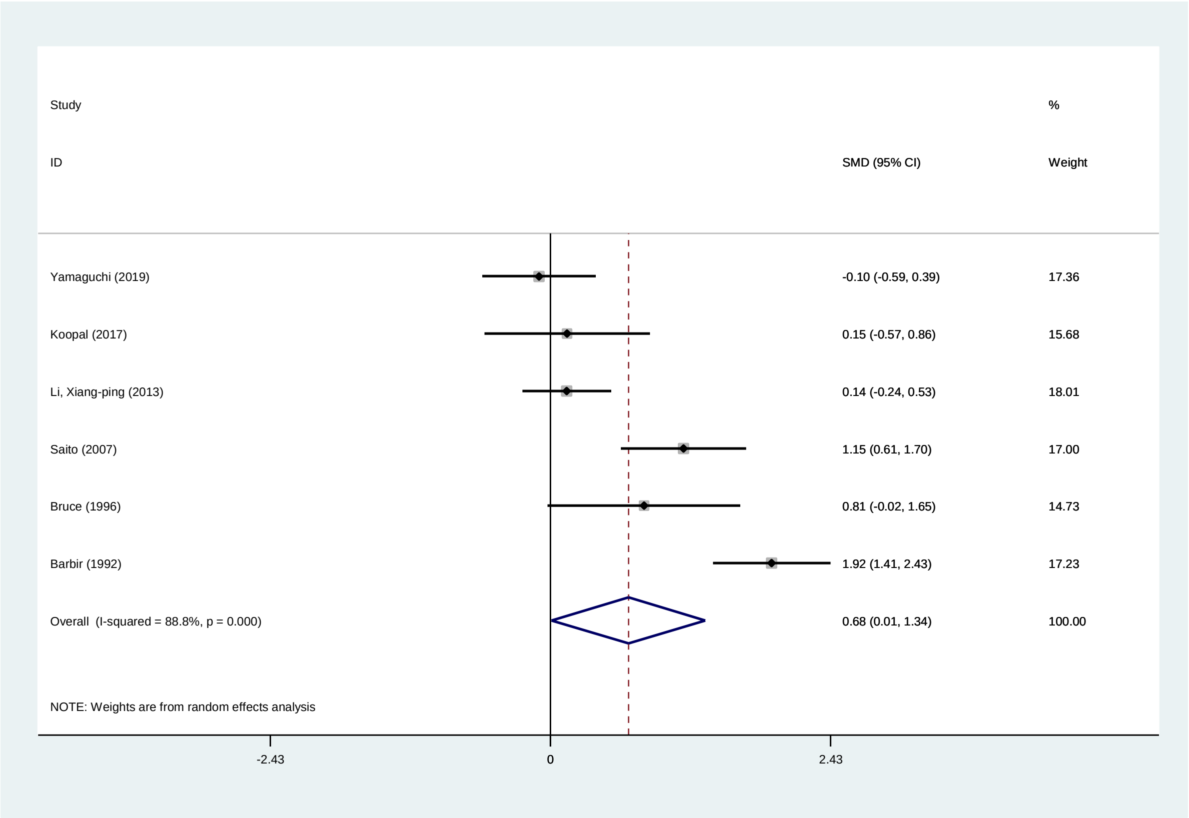

Figure S7: Pooled effects for short term creatinine change (3 months or less) for all studies

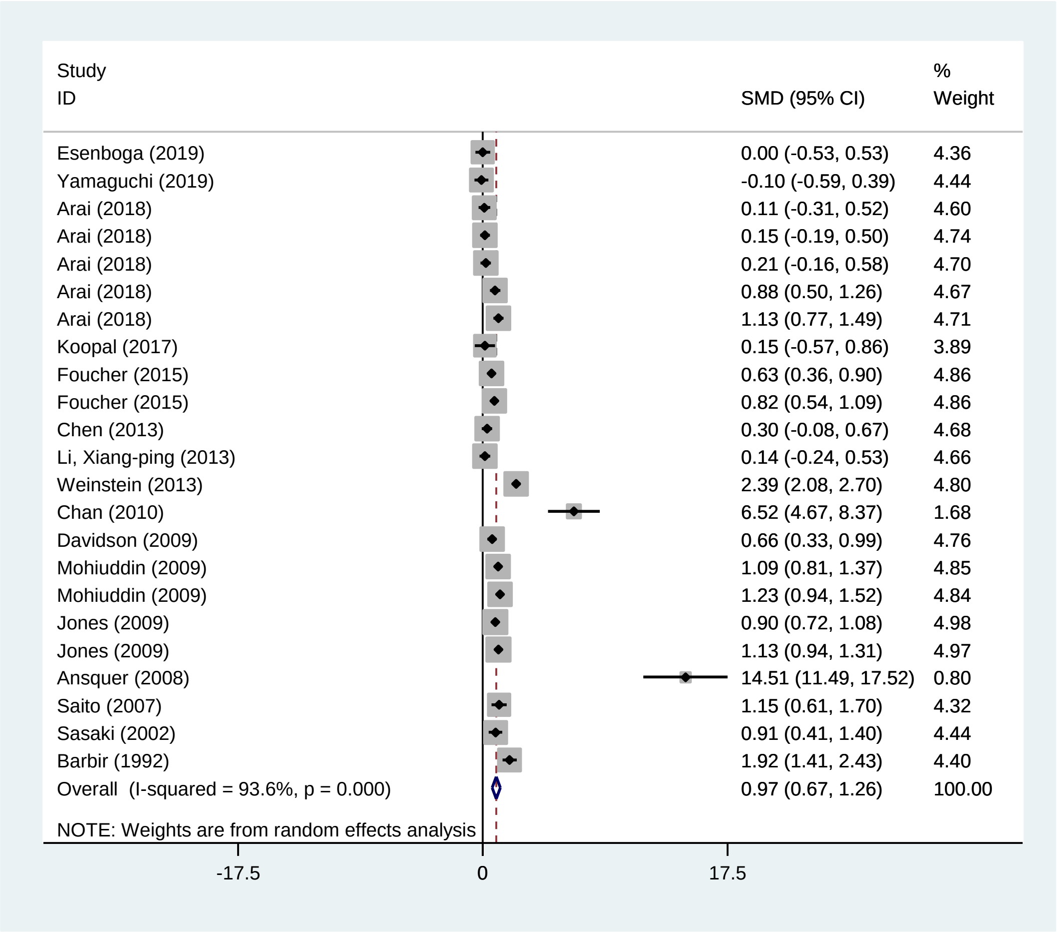

Figure S8: Pooled effects for short term creatinine change for studies examining fenofibrates

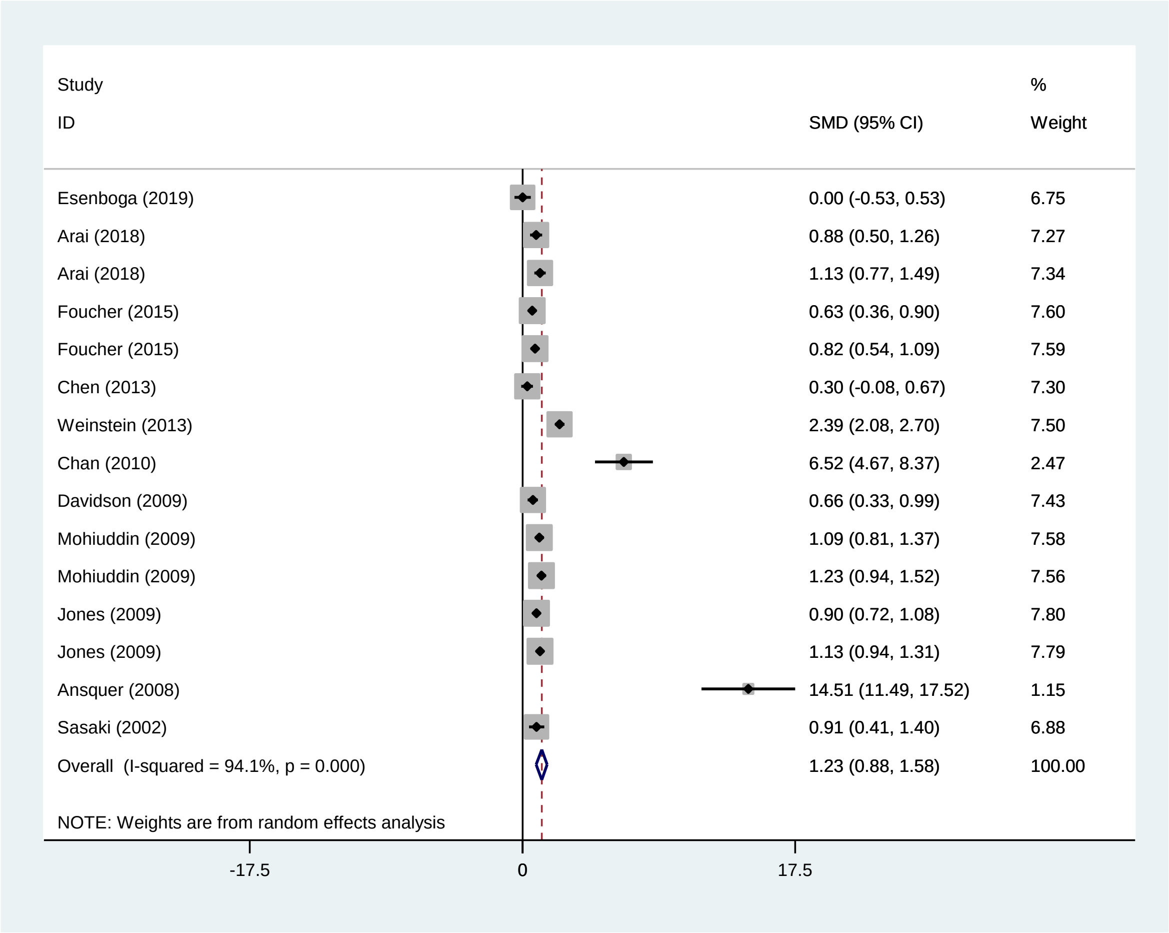

Figure S9: Pooled effects for Short term creatinine changes for studies examining fenofibrate vs placebo

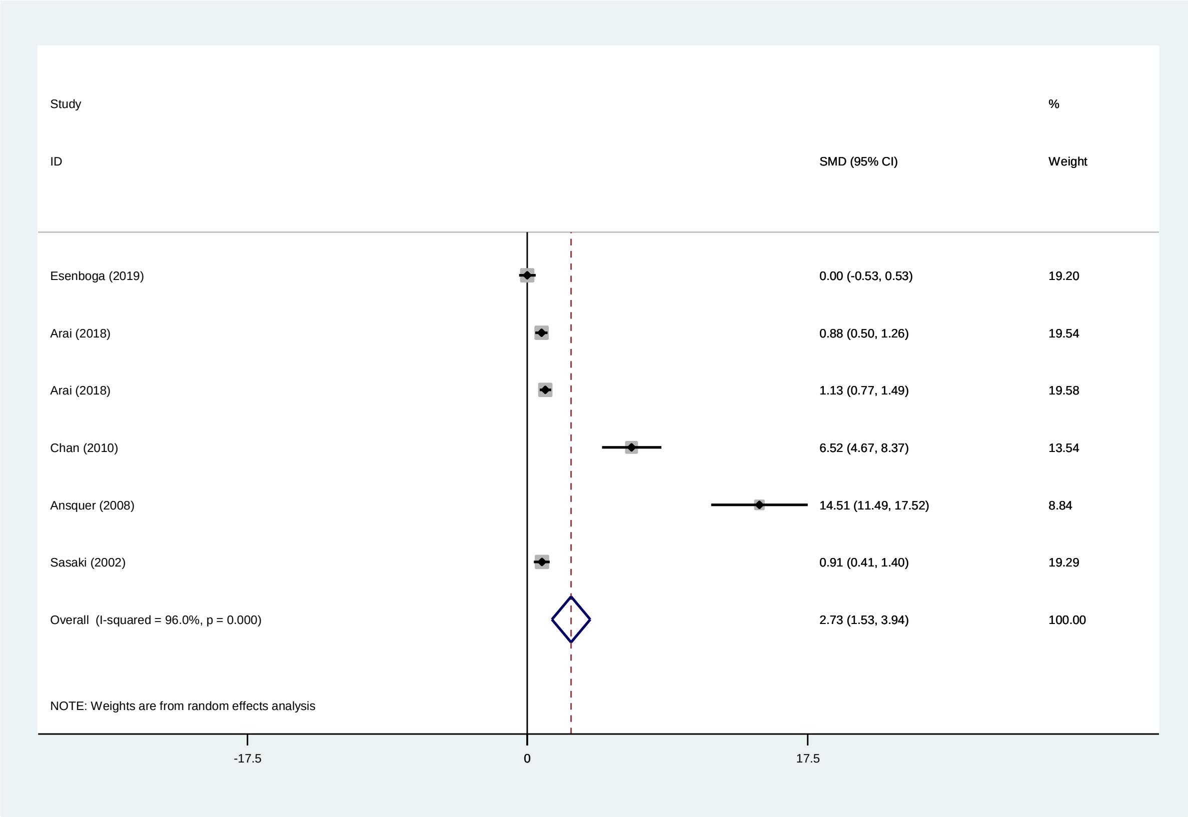

Figure S10: Pooled effects for short term creatinine changes for studies examining fenofibrate plus statin vs statin

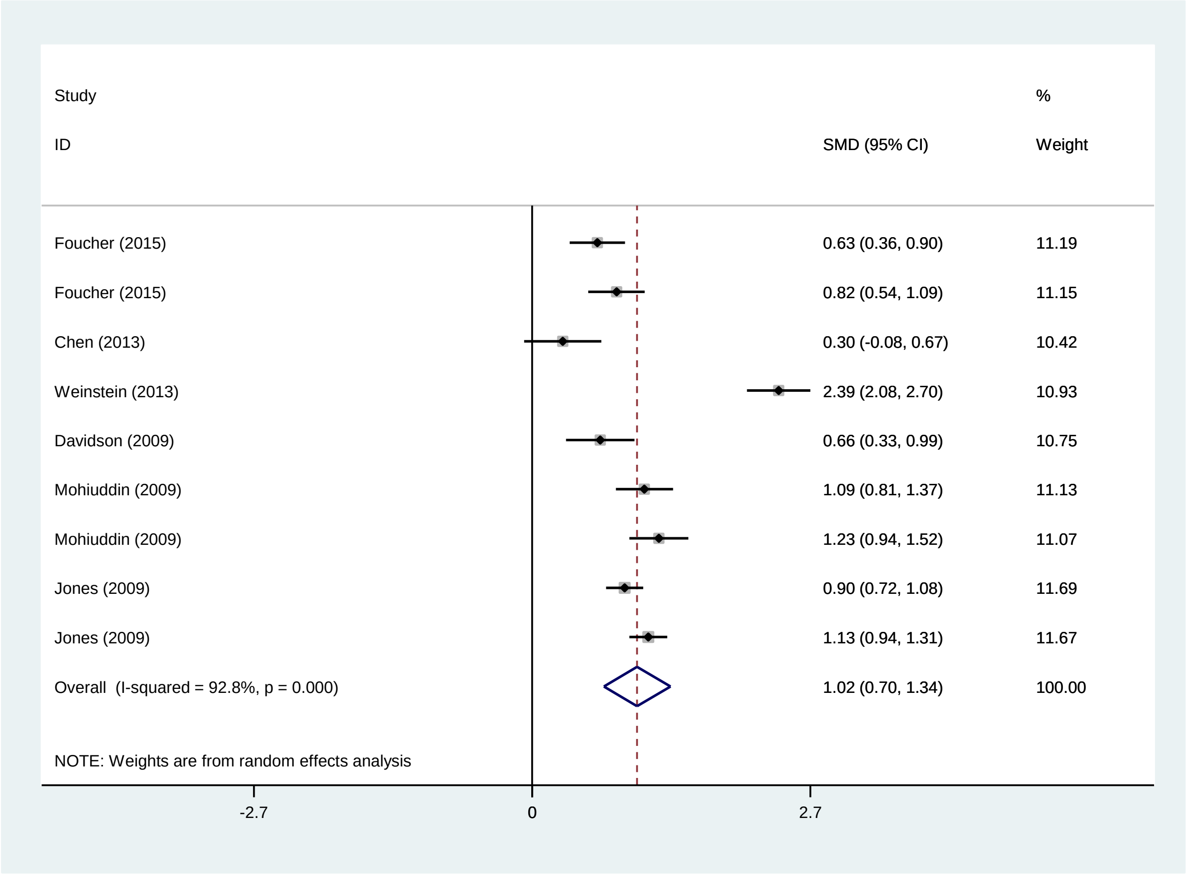

Figure S11: Pooled effects for short term creatinine changes for studies examining Bezafibrates

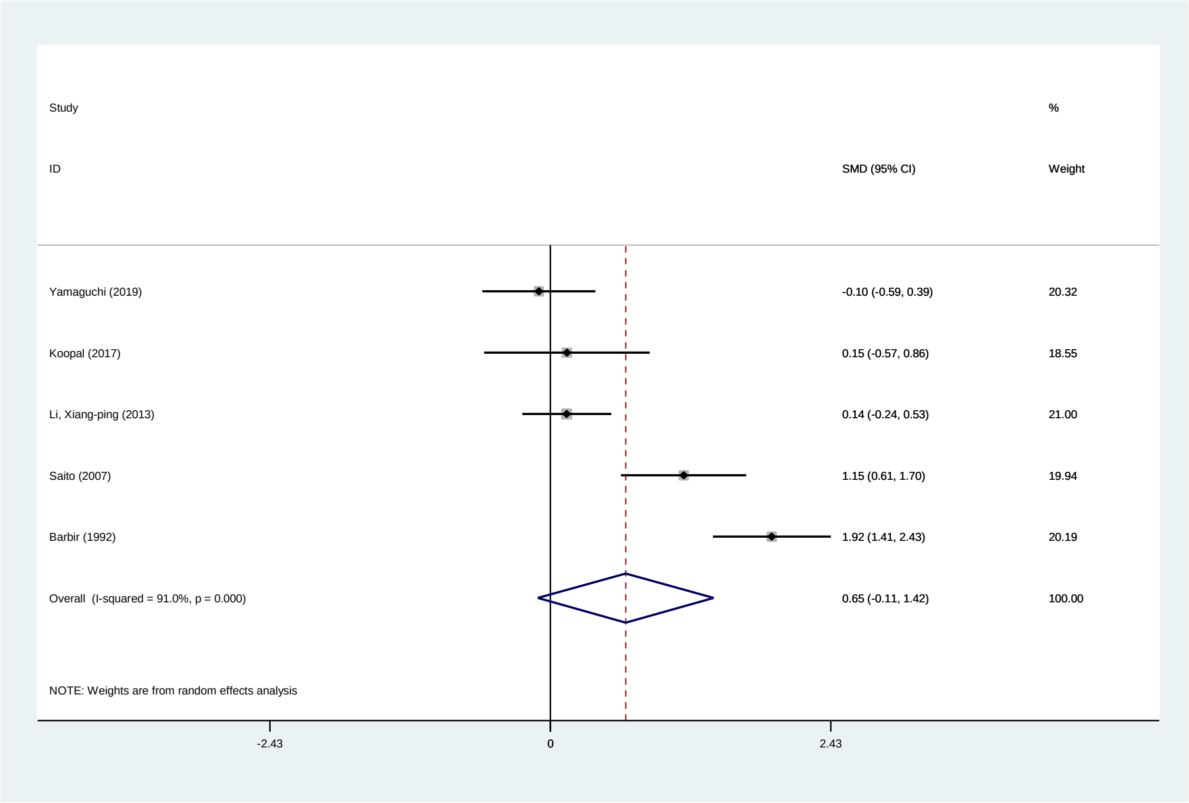

Figure S12: Pooled effects for short term change for studies examining bezafibrate vs placebo

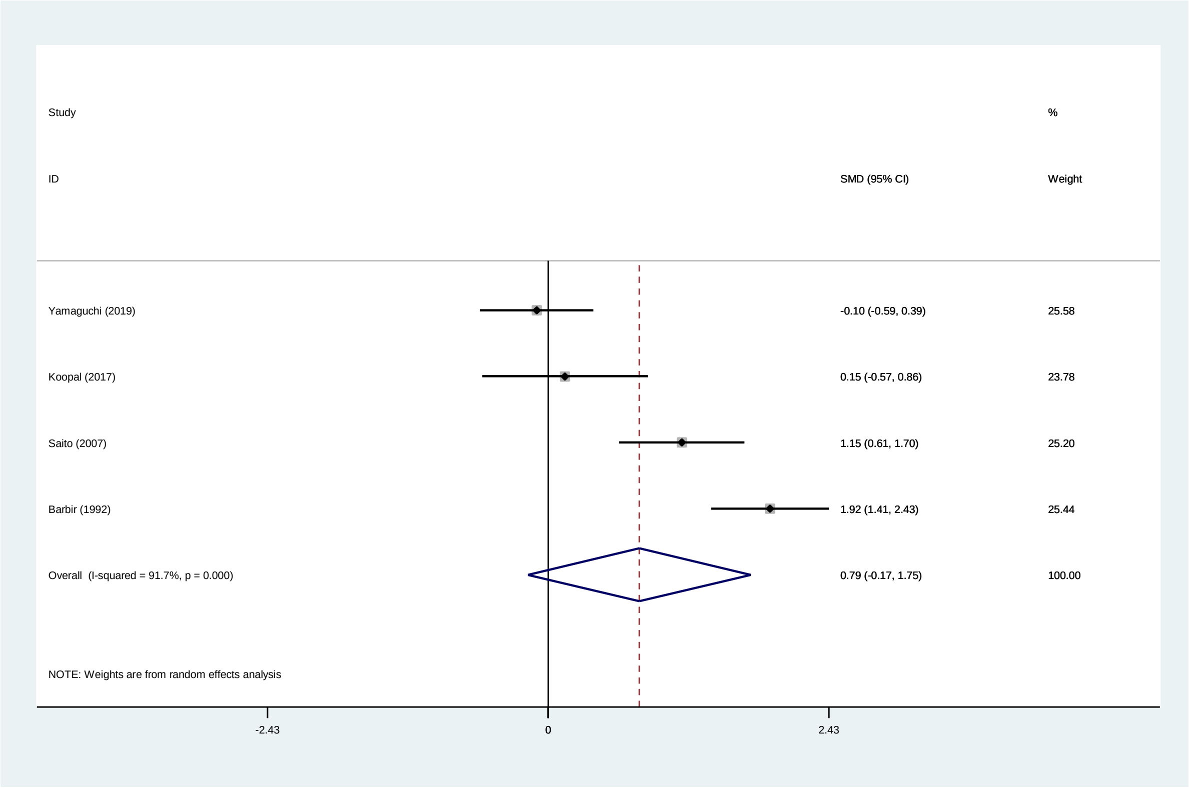

Figure S13: Pooled effects for eGFR for all studies examining fenofibrates

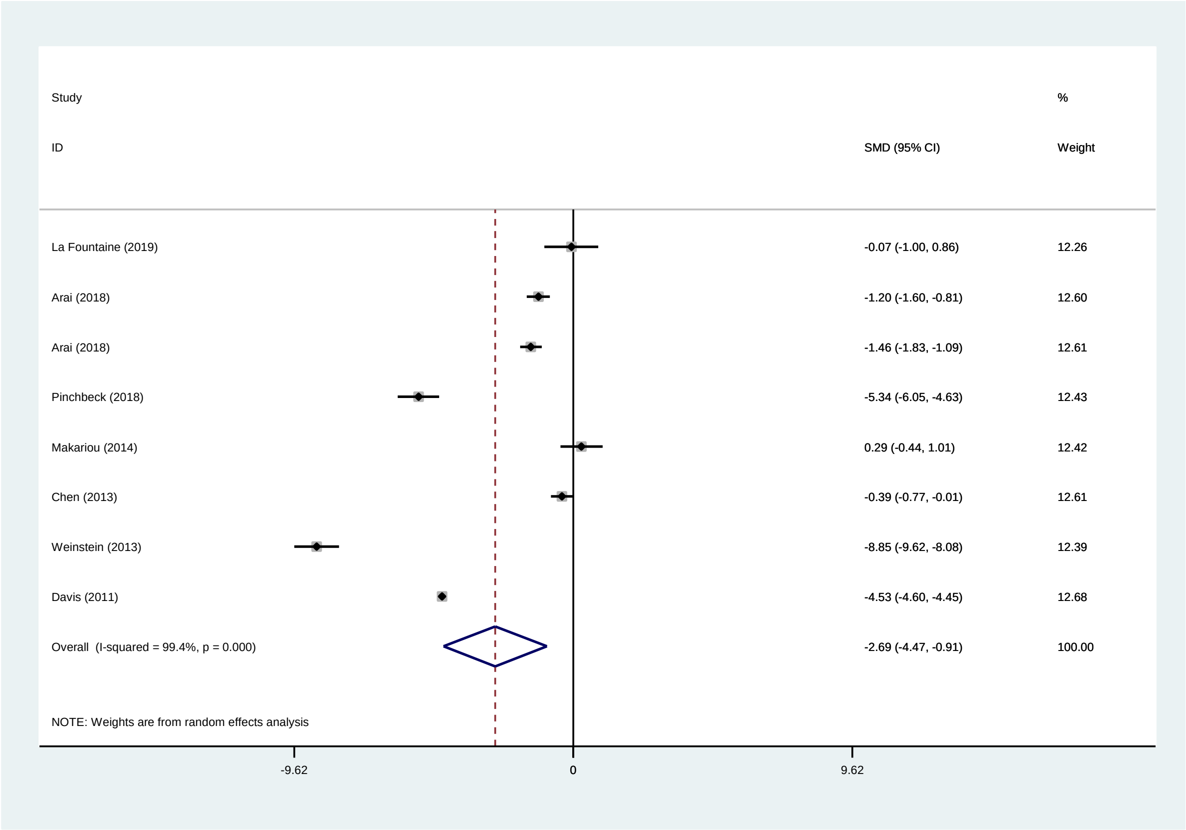

Study
ID
SMD (95% CI)
%
Weight
La Fountaine (2019) -0.07 (-1.00, 0.86) 12.26
Arai (2018) -1.20 (-1.60, -0.81) 12.60
Arai (2018) -1.46 (-1.83, -1.09) 12.61
Pinchbeck (2018) -5.34 (-6.05, -4.63) 12.43
Makariou (2014) 0.29 (-0.44, 1.01) 12.42
Chen (2013) -0.39 (-0.77, -0.01) 12.61
Weinstein (2013) -8.85 (-9.62, -8.08) 12.39
Davis (2011) -4.53 (-4.60, -4.45) 12.68
Overall (I-squared = 99.4%, p = 0.000) -2.69 (-4.47, -0.91) 100.00
NOTE: Weights are from random effects analysis
-9.62
0
9.62

Figure S14: Pooled effects for eGFR for studies examining Fenofibrate vs placebo

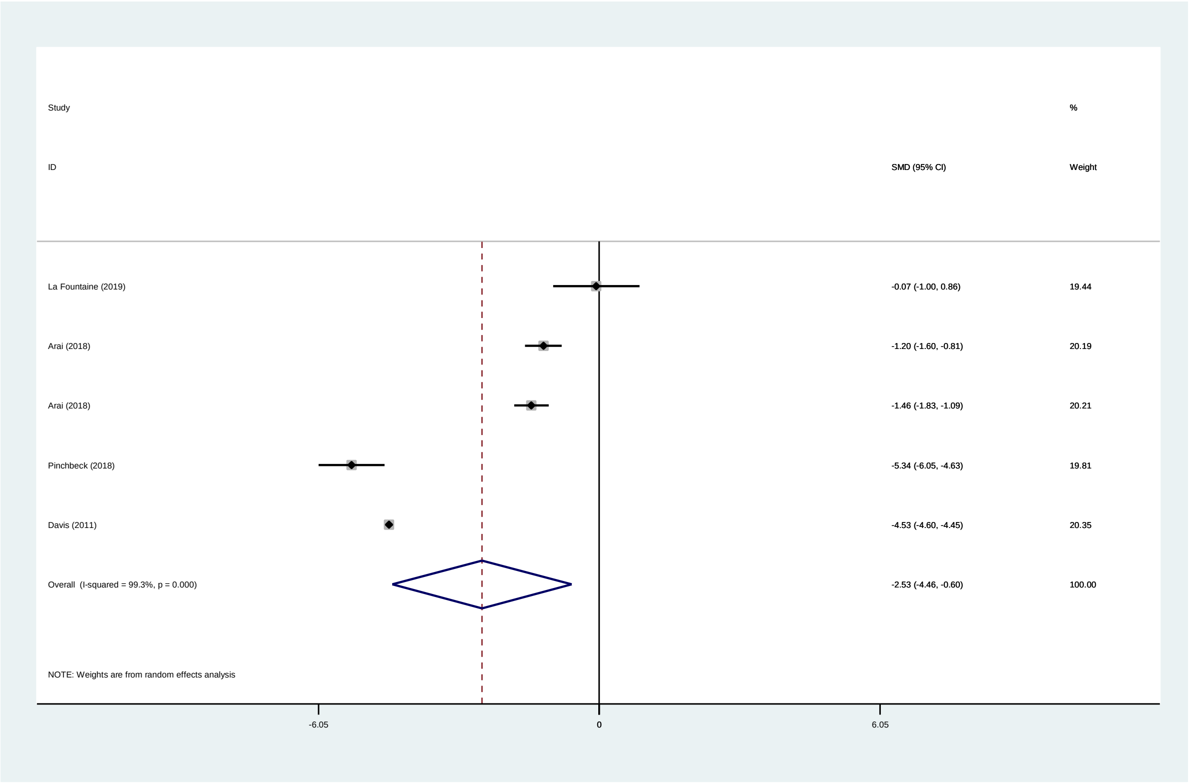

Figure S15: Pooled effects for eGFR for studies examining Fenofibrate + statin vs statin

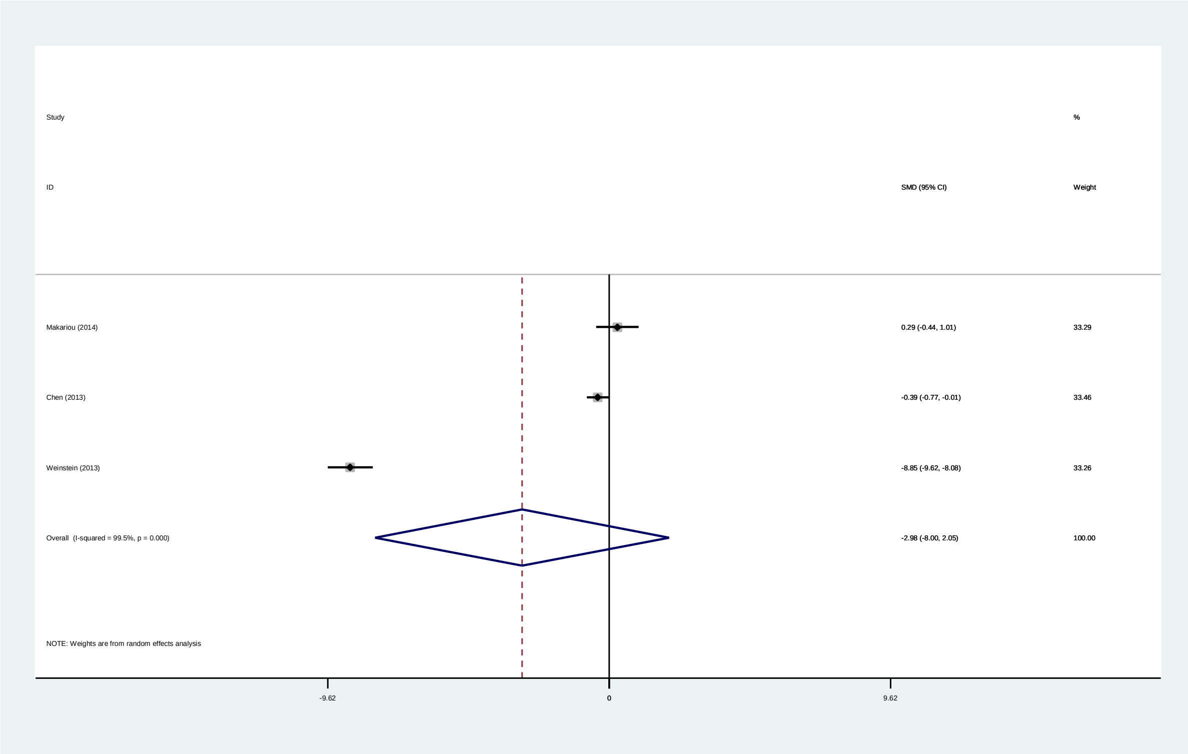

Figure S16: Pooled effects for short term eGFR changes

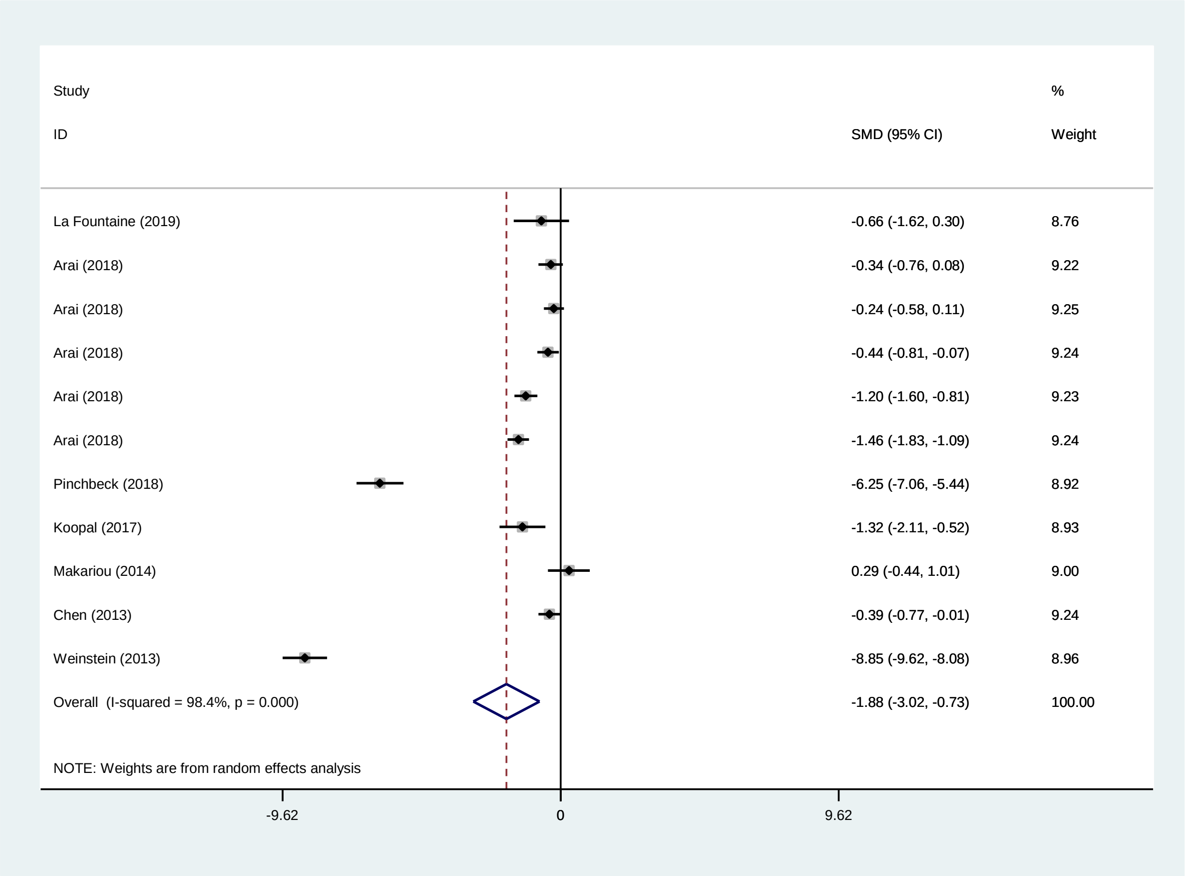

Figure S17: Pooled effects for short term eGFR change for studies examining fenofibrates

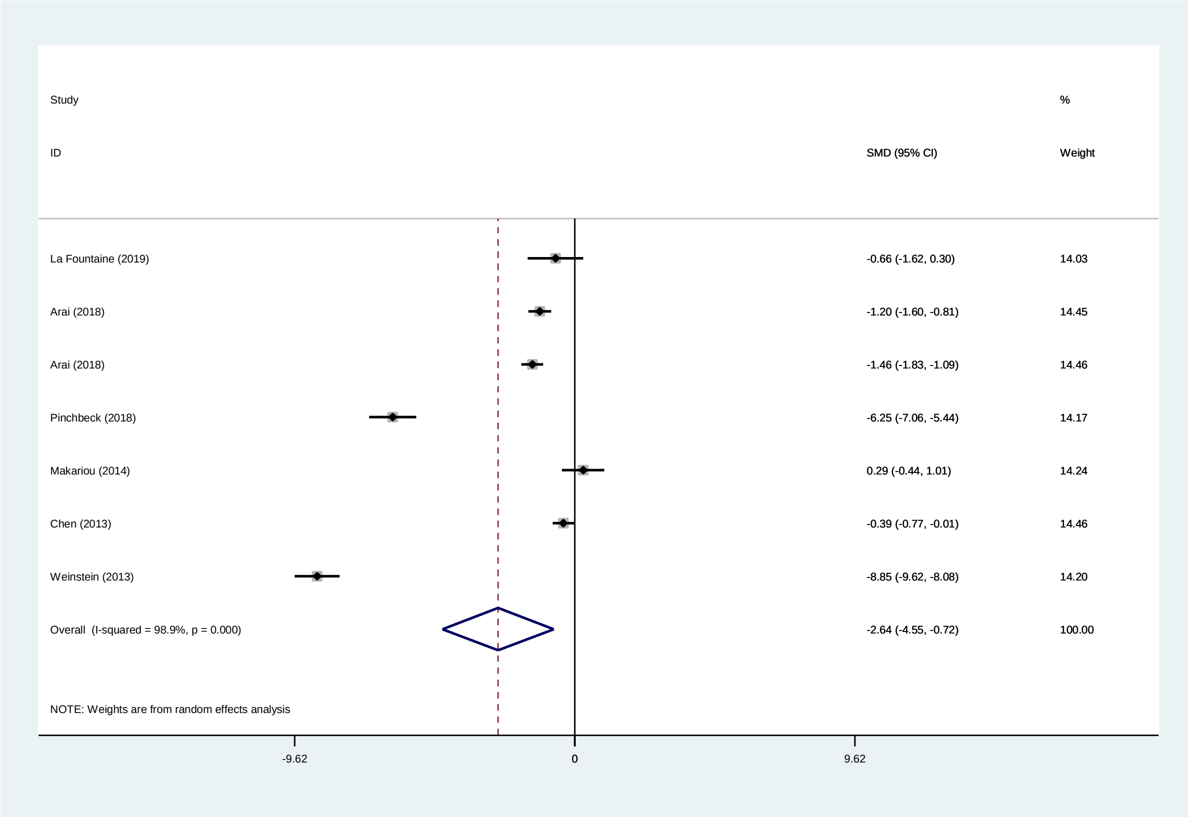

Figure S18: Pooled effects for short term eGFR change for studies examining fenofibrate vs placebo

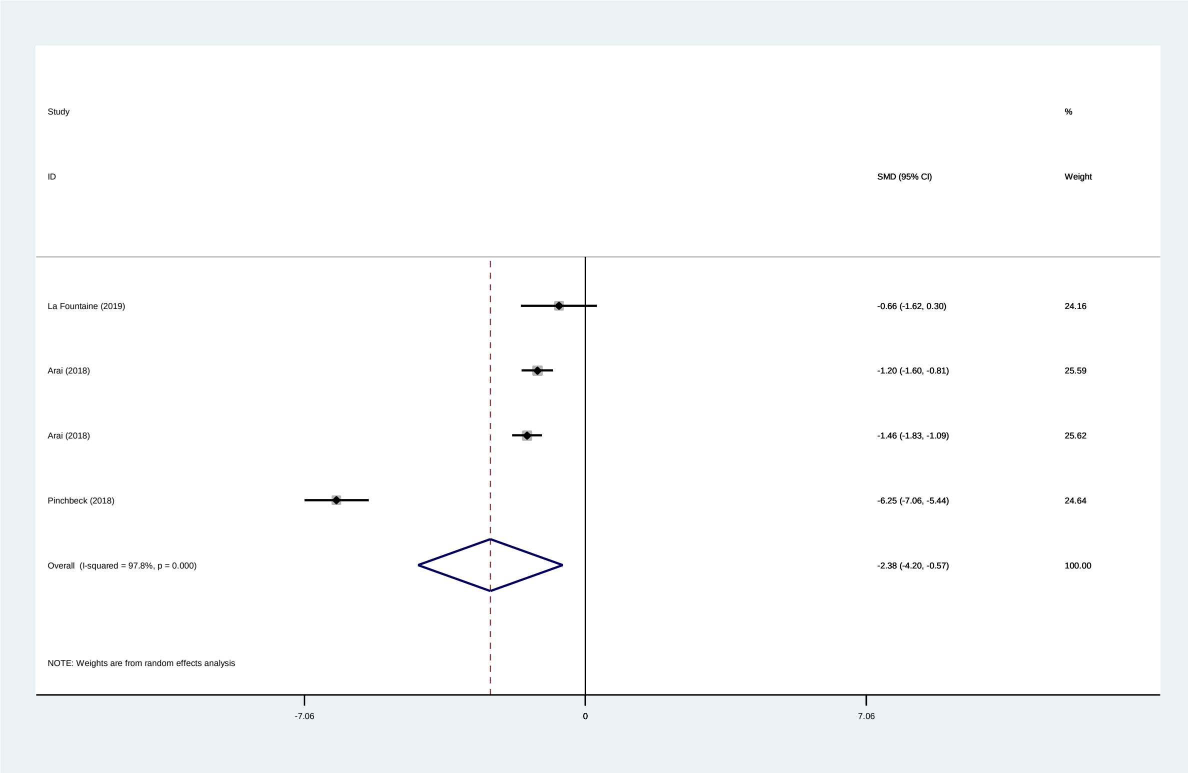

Figure S19: Pooled effects for short term eGFR change for studies examining fenofibrate plus statin vs statin

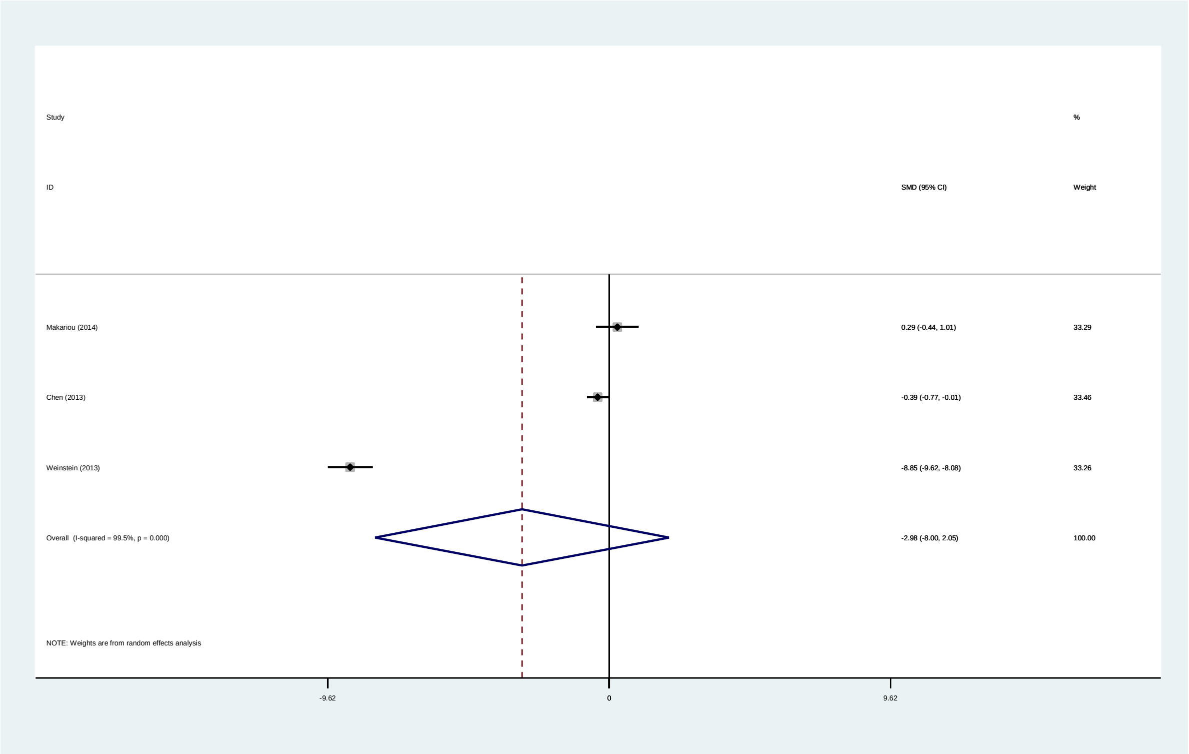

Figure S20: Pooled effects for studies examining urinary protein excretion change

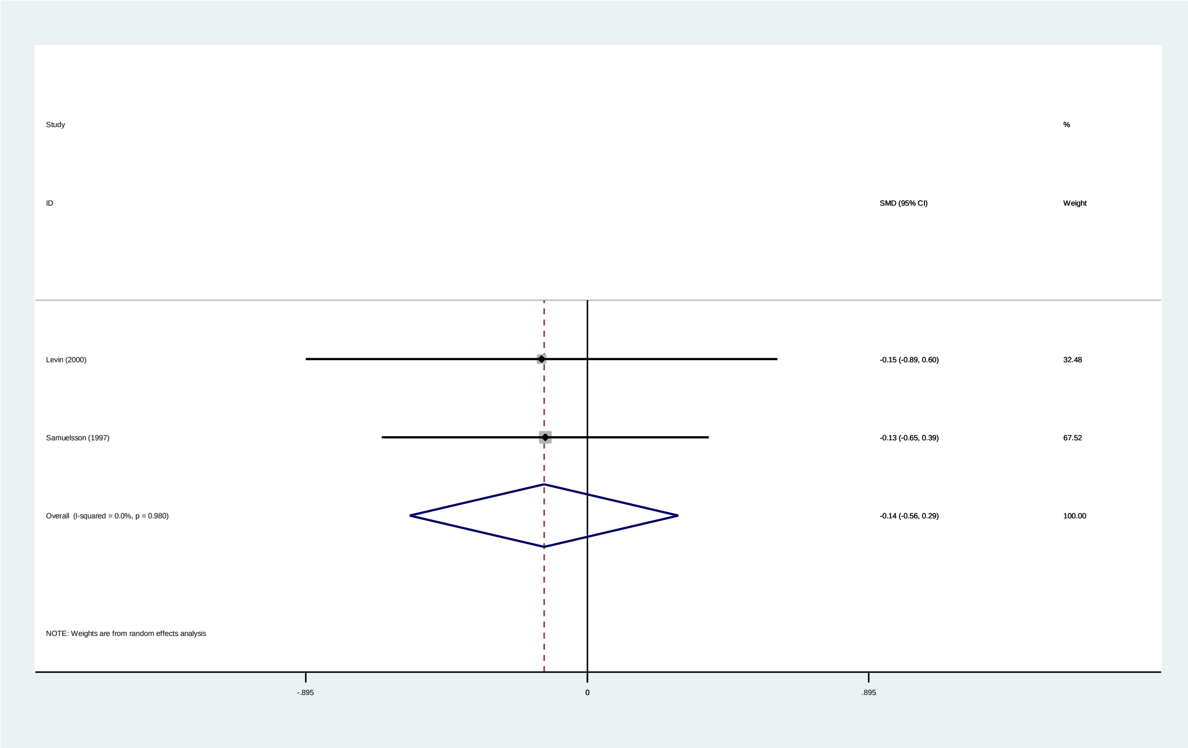
Study
%
ID
SMD (95% CI)
Weight
Levin (2000)
-0.15 (-0.89, 0.60)
32.48
Samuelsson (1997)
-0.13 (-0.65, 0.39)
67.52
Overall (I-squared = 0.0%, p = 0.980)
-0.14 (-0.56, 0.29)
100.00
NOTE: Weights are from random effects analysis
-.895
0
.895

Figure S21: Pooled estimates for End stage kidney disease progression

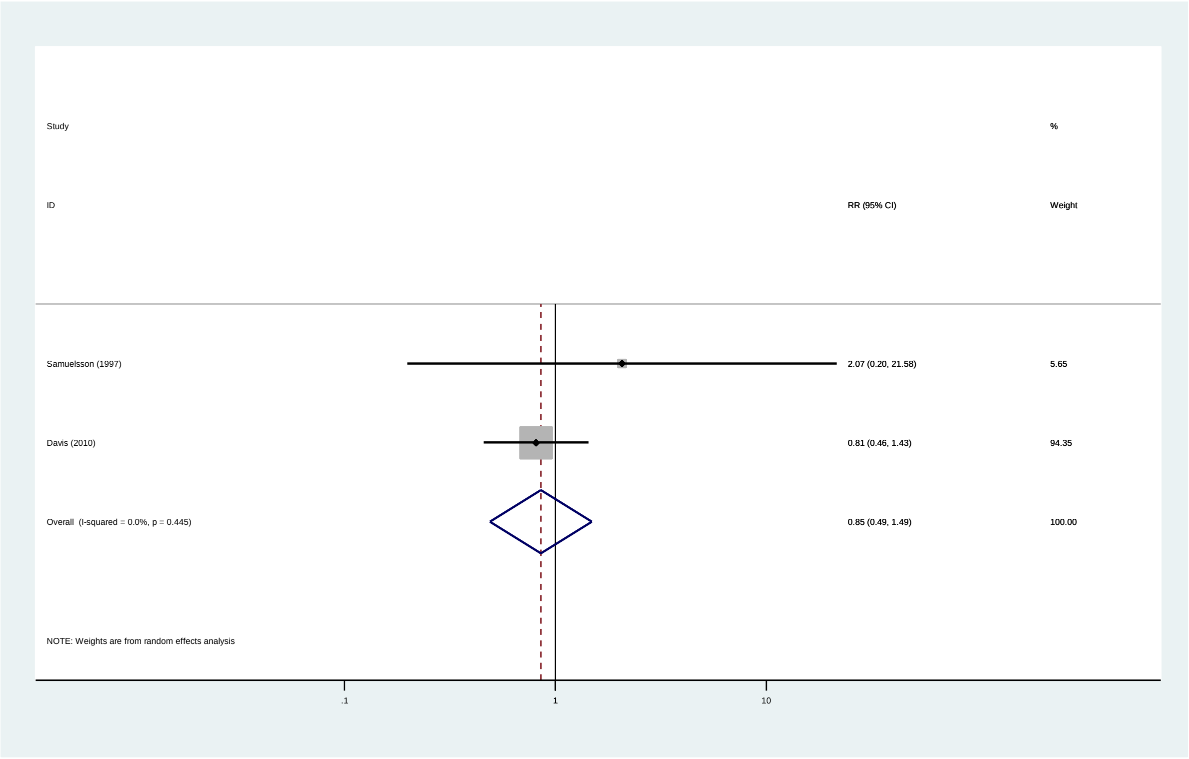

Figure S22: Egger's precision publication bias plot for all studies examining creatinine changes.

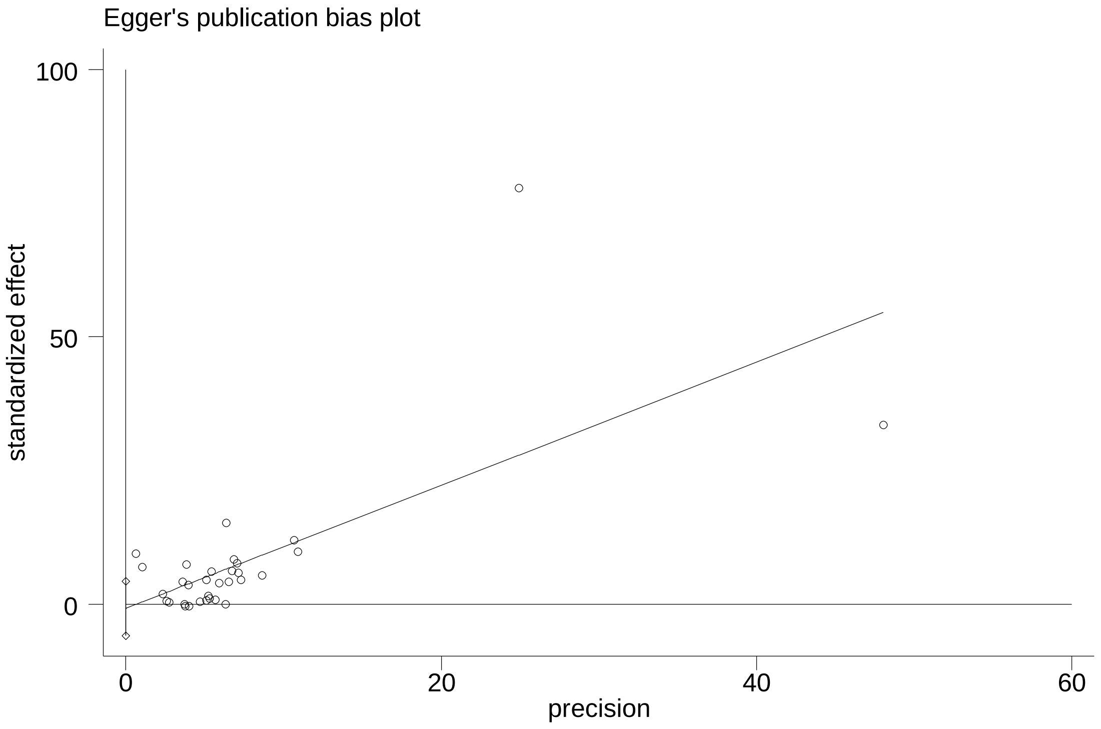

Figure S23: Egger's precision publication bias plot for creatinine changes in studies using fenofibrates.

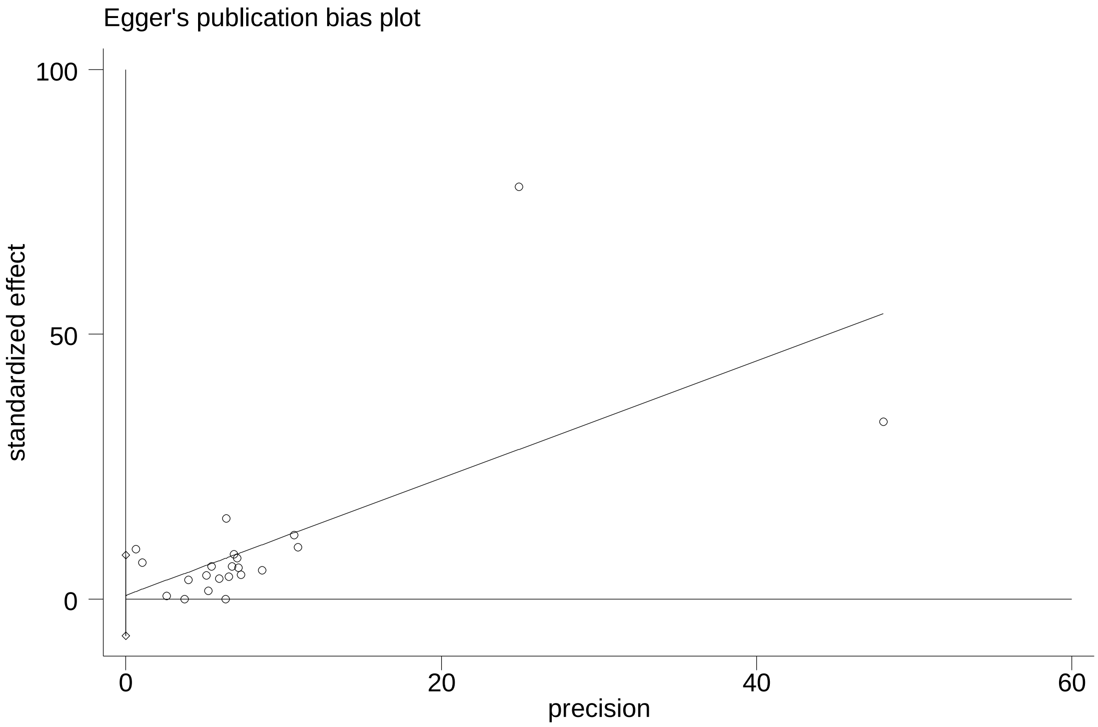

Figure S24: Egger’s precision publication bias plot for creatinine changes studies examining fenofibrate + statin vs statin.

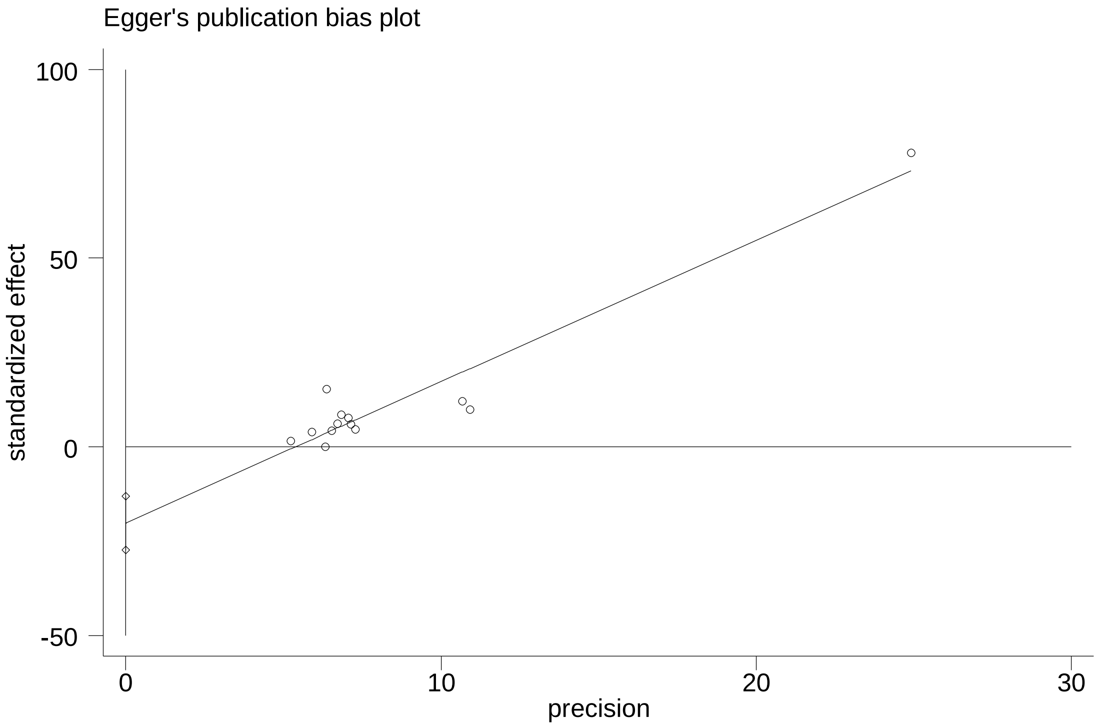

Figure S25: Egger's precision publication bias plot for short term creatinine change

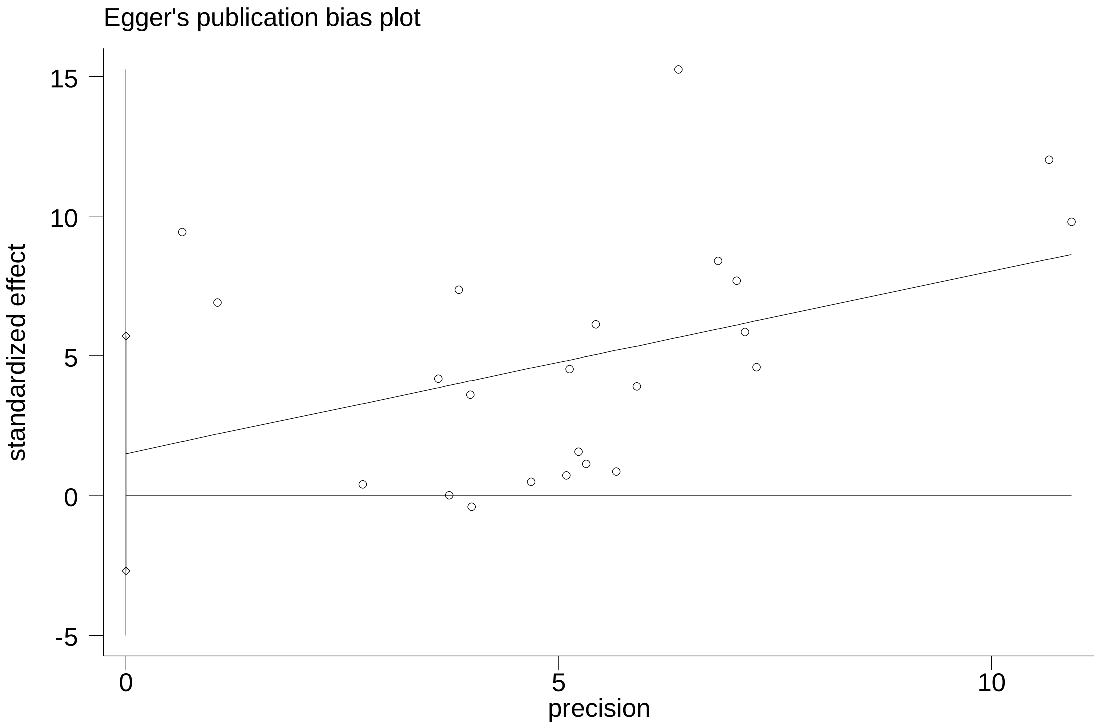

Figure S26: Egger's precision publication bias plot for all studies examining change in eGFR

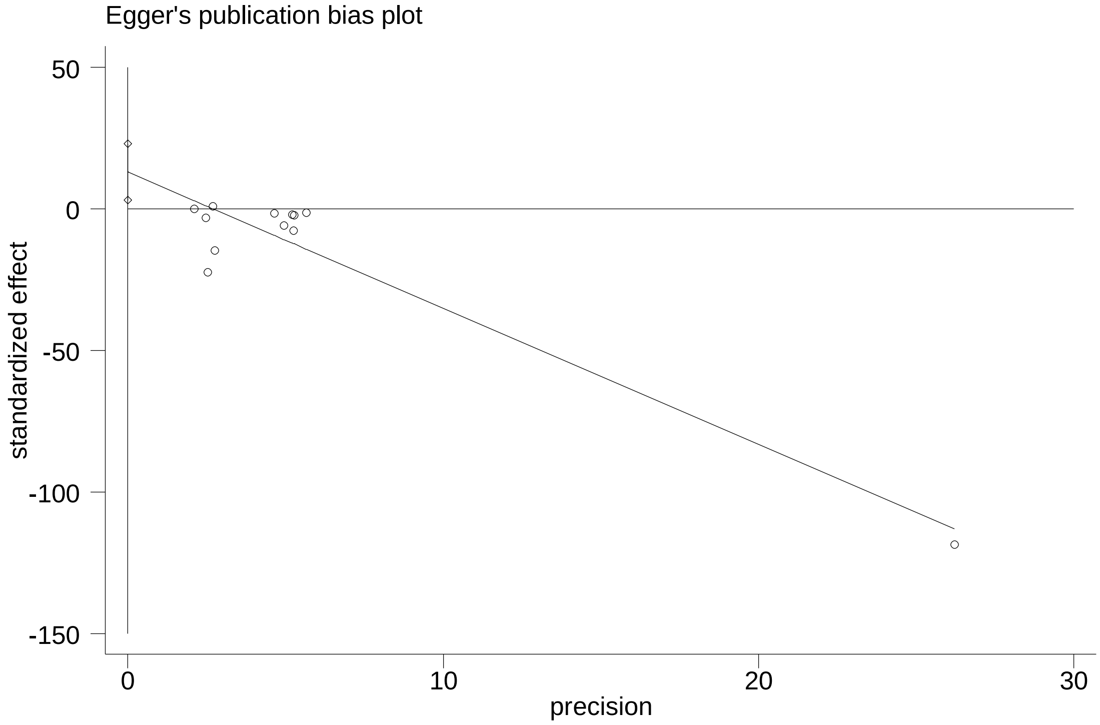

Table S1: Patient characteristics

1st author	Year	Study name	CKD	Diabetes Melitus type 2		Baseline Creatinine		Baseline eGFR		Follow up
						I	C	I	C	
Esenboga	2019		No	No		0.83 (0.68-0.98)	0.74 (0.65–0.92)	NR		12 weeks
La Fountaine	2019		No	No		NR		121 (106 – 135)	126 (107-144)	1, 2, 3, 4 months
Yamaguchi	2019		No	100%		0.80 ± 0.22	0.85 ± 0.37	NR		12 weeks
Arai	2018		No	For Pemafibrate 0.1mg (8.9%), 0.2mg (18.8%), 0.4mg (17.9%). For Fenofibrate 100mg (12.9%), 200mg (13.6%) For Placebo 16.3%.		For Pemafibrate 0.1mg (0.86±0.13), 0.2mg (0.81±0.17), 0.4mg (0.83±0.15). For Fenofibrate 100mg (0.8±0.14), 200mg (0.83±0.13) For Placebo 0.81±0.13		For Pemafibrate 0.1mg (76±13), 0.2mg (79.5±17), 0.4mg (79±15). For Fenofibrate 100mg (81±14), 200mg (77±13) For Placebo 78±12		12 weeks
Pinchbeck	2018	FAME	No	32.9%	25.7 %	0.95 (0.81-1.17)	0.97 (0.84 - 1.11)	69.5 (60.0 – 88.0)	73.5 (63.5 - 86.3)	5, 10, 15, 20, 25 weeks
Koopal	2017		No	27%		0.86 ± 0.15		85 ± 7		6 weeks, 2 weeks washout, crossover, 6 weeks follow up, 2 weeks washout
Foucher	2015		No	Fenofibrate145 + simvastatin 20mg (55%), Fenofibrate + 145+ simvastatin 40mg (48%), Simvastatin 20mg (64%), simvastatin 40% (49%)		Fenofibrate145 + simvastatin 20mg (0.90±0.18), Fenofibrate + 145+ simvastatin 40mg (0.88±0.18), Simvastatin 20mg (0.89±0.18), simvastatin 40% (0.94±0.34)		NR		6 and 12 weeks
Makariou	2014		No	No		Controlled patients but they		95 ± 29	86 ± 28	12 weeks

						don't report number				
Chen	2013		Included patients with baseline Cr < 2mg/dl	All patients had T2DM or metabolic syndrome. Authors don't report numbers		1.29 ±0.36	1.11± 0.34	60.73 ± 18.20	69.47 ± 13.23	12 weeks
Li Xiang-ping	2013		No	No		0.97 ±0.21	0.99 ±0.25	NR		12 weeks
Weinstein	2013		eGFR between 30–59	59.3 %	55.7%	1.36	1.38	48.7 7	47.08	4, 8, 12, 16 and 24 weeks
Lee	2012		Included patients with baseline Cr <1.5 mg/dl	6.7 %	10%	0.86 ± 0.25	0.82 ± 0.19	NR		24 weeks
Davis	2011	FIELD	Included patients with baseline Cr < 130 µmol/l,	100%		0.88 ± 0.18	0.87 ± 0.18	87.6± 18.5	87.8 ± 18.3	3, 12, 24, 36, 48, 54 months
Ginsberg	2010	ACCO RD	eGFR > 50: 97.4% for Fenofibrate and 97.5% for Placebo. eGFR 30-49: 2.6% for Fenofibrate and 2.5% for placebo	100%		0.9 ±0.2 both groups		eGFR > 50: 97.4% for Fenofibrate and 97.5% for Placebo. eGFR 30-49: 2.6% for Fenofibrate and 2.5% for placebo		4, 8, 12, 24, 36, 48, 60, 72, 84 months
Chan	2010		Included patients with baseline serum creatinine <150 µmol/l;	100%		0.90 ± 0.03	0.93 ± 0.03	NR		12 weeks, crossover, 4 weeks washout between interventi ons
Derosa	2009		Normal baseline serum creatinine level for age and sex, no history of albuminuria or nephrotic syndrome	100%		0.99 ± 0.09	1.03 ± 0.12	NR		6 and 12 months
Davidson	2009		Patients with recent history (6 months) of significant renal disease were excluded	Included only controlled T2DM, authors don't mention number of patients		1.0 ± 0.2	0.9 ± 0.28	NR		4, 8, 12 weeks
Mohiuddin	2009		NR	Fenofibric acid +		Fenofibric acid + simvastatin		NR		12 weeks

				simvastatin 20mg (21.8%), simvastatin 20mg (21.8%), fenofibric acid + simvastatin 40mg (24.6%), simvastatin 40mg (24.6%)		20mg (0.90), simvastatin 20mg (0.90), fenofibric acid + simvastatin 40mg (0.88), simvastatin 40mg (0.89)			
Jones	2009		No	19.9% each arm		NR		NR	12 weeks
Ansquer	2008		No	No		0.84 ± 0.11	0.85 ± 0.12	NR	12 weeks
Saito	2007		Patients with baseline serum creatinine >1.5 mg/dL were excluded	3.7 %	5.7%	0.804 ± 0.18	0.759 ± 0.18	NR	8 weeks
Ansquer	2005	DAIS	Included patients had not significant renal disease, shown by a history of proteinuria and/or serum creatinine levels of 1.7 mg/dL or greater.	100%		1.0 ± 0.2	0.9 ± 0.1	NR	Annually, for at least 3 years
Athyros	2005		Patients with baseline serum creatinine >1.5 mg/dl were excluded	No		1.05 ±0.16	1.04 ±0.15	NR	12 months
Sasaki	2002		Patients with severe kidney disease, including nephrotic syndrome, were excluded	Patients with controlled T2DM were included but authors do not report numbers		0.71 ± 0.17		NR	8 weeks and then crossover
Levin	2000		Included patients had creatinine clearance 20 --	50%	58.3%	2.10 ± 0.81	2.15 ± 1.08	NR	2, 46 months

			74 ml/min/1.73 m^2							
Samuelsson	1997		Included patients with GFR of 10-70 ml/min x1.73m2 BSA	No		3.37 ± 1.53	4.21 ± 2.12	36.2 ± 12.9	34.8 ± 18.6	6 and 12 months
Bruce	1996		mild renal impairment (creatinine < 145 μmol/L),	100%		1.94 ± 0.3	1.84 ± 0.19	91±49 average for all		6 months
Barbir	1992		Patients with serum creatinine >180 μmol/L) were excluded	No		1.58	1.57	NR		3 months
Jones	1990		all had normal plasma creatinine	100%		0.93 ±0.05 for all		NR		3 months

Abbreviations: CKD – Chronic kidney disease; I – intervention; C – Comparator; NR – Not reported; T2DM – Type 2 Diabetes Mellitus; Cr – Creatinine;

Appendix

A1 - Search algorithm:

(fibrate OR clofibrate OR "clofibric acid" OR bezafibrate OR gemfibrozil OR fenofibrate OR procetofen) AND ("Mortality"[MeSH] OR mortality OR "Cardiovascular Diseases"[MeSH] OR "cardiovascular diseases" OR "Kidney Diseases"[MeSH] OR "Kidney Diseases" OR "Renal Replacement Therapy"[MeSH] OR "Renal Replacement Therapy" OR "Kidney Function Tests"[MeSH] OR "Kidney Function Tests" OR "Kidney"[MeSH] OR kidney OR "Glomerular Filtration Rate"[MeSH] OR "Glomerular Filtration Rate" OR "Proteinuria"[MeSH] OR Proteinuria)
